# Heteroleptic samarium(iii) halide complexes probed by fluorescence-detected L_3_-edge X-ray absorption spectroscopy†
†Electronic supplementary information (ESI) available: Research data files supporting this publication are available from Mendeley Data at DOI: 10.17632/7jkwgy4bfr.1. CCDC 1833620–1833623 for the solid-state structures of **2-X**. For the ESI and crystallographic data in CIF or other electronic format see DOI: 10.1039/c8dt01452c


**DOI:** 10.1039/c8dt01452c

**Published:** 2018-05-04

**Authors:** Conrad A. P. Goodwin, Benjamin L. L. Réant, Jon G. C. Kragskow, Ida M. DiMucci, Kyle M. Lancaster, David P. Mills, Stephen Sproules

**Affiliations:** a School of Chemistry , The University of Manchester , Oxford Road , Manchester M13 9PL , UK . Email: david.mills@manchester.ac.uk; b Department of Chemistry and Chemical Biology , Baker Laboratory , Cornell University , Ithaca , New York , 14853 , USA . Email: kml236@cornell.edu; c WestCHEM , School of Chemistry , The University of Glasgow , Glasgow G12 8QQ , UK . Email: stephen.sproules@glasgow.ac.uk

## Abstract

The addition of various oxidants to the near-linear Sm(ii) complex [Sm(N^††^)_2_] (**1**), where N^††^ is the bulky bis(triisopropylsilyl)amide ligand {N(Si^*i*^Pr_3_)_2_}, afforded a family of heteroleptic three-coordinate Sm(iii) halide complexes, [Sm(N^††^)_2_(X)] (X = F, **2-F**; Cl, **2-Cl**; Br, **2-Br**; I, **2-I**). In addition, the trinuclear cluster [{Sm(N^††^)}_3_(μ_2_-I)_3_(μ_3_-I)_2_] (**3**), which formally contains one Sm(ii) and two Sm(iii) centres, was isolated during the synthesis of **2-I**. Complexes **2-X** are remarkably stable towards ligand redistribution, which is often a facile process for heteroleptic complexes of smaller monodentate ligands in lanthanide chemistry, including the related bis(trimethylsilyl)amide {N(SiMe_3_)_2_} (N′′). Complexes **2-X** and **3** have been characterised by single crystal X-ray diffraction, elemental analysis, multinuclear NMR, FTIR and electronic spectroscopy. The Lα_1_ fluorescence-detected X-ray absorption spectra recorded at the Sm L_3_-edge for **2-X** exhibited a resolved pre-edge peak defined as an envelope of quadrupole-allowed 2p → 4f transitions. The X-ray absorption spectral features were successfully reproduced using time-dependent density functional theoretical (TD-DFT) calculations that synergistically support the experimental observations as well as the theoretical model upon which the electronic structure and bonding in these lanthanide complexes is derived.

## Abstract 2

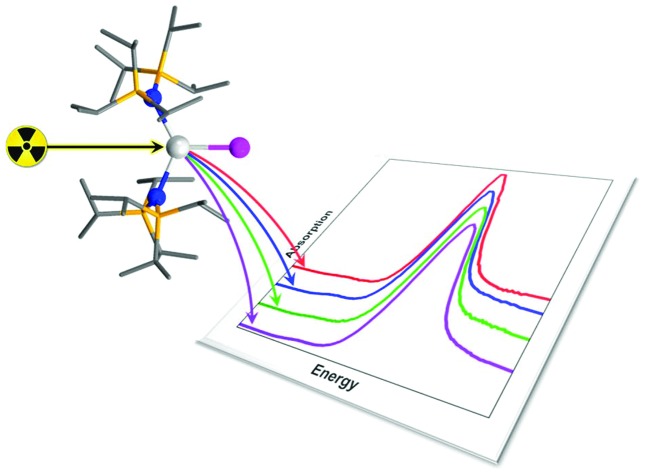
The novel series of heteroleptic Sm(iii) halide complexes provides the backdrop for a fluorescence-detected Lα_1_ X-ray absorption spectroscopic study.

## Introduction

Low-coordinate metal complexes can exhibit remarkable physicochemical properties, and amide ligands have proved particularly effective in stabilising such motifs for lanthanides (Ln).^1^ This is exemplified by the pioneering work of Bradley and co-workers, who reported the first three-coordinate f element complexes [Ln(N′′)_3_] (N′′ = {N(SiMe_3_)_2_}).^2^ Inspired by this previous work, some of us have developed sterically encumbered bis(silyl)amide ligands that have led to the synthesis of novel low-coordinate Ln complexes.^3^–^7^ Silylamides are well known to stabilise complexes in both Ln and actinide chemistry;^8^ the most relevant to this work are the [Ln(N^††^)_2_] (Ln = Sm, **1**; Eu, Tm, and Yb; N^††^ = {N(Si^*i*^Pr_3_)_2_}) series which represent the first examples of near-linear f element complexes.^3^,^5^ We performed a preliminary reactivity study of **1** with TEMPO (2,2,6,6-tetramethyl-piperidinyl-1-oxy), benzophenone and azobenzene, and found that despite the considerable steric demands of N^††^ an extra ligand could be accommodated into the Sm(iii) coordination sphere upon oxidation.^5^ Here we show that **1** reacts with various oxidants to give a structurally homologous family of heteroleptic Sm(iii) bis(silyl)amide halide complexes. Similar methodologies have recently been applied by Anwander and co-workers to generate Ln(iii) halide complexes from Ln(ii) bis(silylamide) precursors.^9^

The utility of this family of compounds, where the identity of the coordinated halide is the single variable, makes them the ideal series to probe the X-ray spectroscopy at the Sm L_3_-edge. Whilst there is a substantial body of work that has employed X-ray absorption spectroscopy (XAS) in the analysis of Sm in solid materials,^10^–^12^ primarily at the more accessible L_3_-edge, there is a paucity of studies on molecular systems. To the best of our knowledge, there are just two.^13^,^14^ The most recent work from Batista, Evans, Kozimor and co-workers presented an L-edge XAS study of low-coordinate Ln(ii) and Ln(iii) (Ln = Pr, Nd, Sm, Gd, Tb, Dy, Ho, Er, Tb, and Lu) complexes that elegantly pinpointed these low-valent (+ii) ions that possess a 4f^*n*^ electron configuration and others with the rather unexpected 4f^*n*–1^5d^1^ electron configuration;^14^ the latter exhibited extraordinary magnetic properties.^15^ XAS is particularly advantageous for such a study because it is element specific, and arguably the most direct experimental method for evaluating the effective nuclear charge (oxidation level) of the element under examination.^16^,^17^ Specific to the aforementioned Ln compounds, the L-edge spectra gave well-separated transitions from the 2p level to the 4f and 5d acceptor levels that were shown to be sensitive to the electronic population of the 5d orbitals and therein cleanly diagnostic of the electron configuration. In addition to the energy position of the spectral features, intensities provide critical insight into the electronic structure and bonding within the system. This has been heavily exploited across a wide range of disciplines from bioinorganic chemistry to heterogeneous catalysis.^18^ Insight into the bonding within Ln complexes is not readily forthcoming from XAS studies because the spectra involving excitation from the 2p level suffer from core-hole lifetime broadening that obscures the features that directly probe the 4f orbitals – the partially filled valence shell that gives these metal ions their distinctive physical properties.^19^ However, the dipole forbidden, quadrupole-allowed 2p → 4f transitions are better resolved using the fluorescence detection of the Lα_1_ emission line where the core-hole lifetime broadening is significantly attenuated, giving rise to X-ray absorption spectra with narrower linewidths.^17^,^20^ This method – high-energy resolution fluorescence-detected (HERFD) XAS – was premiered by Hämäläinen *et al.* who unearthed elusive pre-edge features in the Dy L_3_-edge spectrum of Dy(NO_3_)_3_.^21^ Recent advances in instrument optics and greater access to synchrotron facilities globally have prompted a surge in HERFD-XAS to study the K pre-edge of first-row transition metal complexes,^22^,^23^ K- and L-edges of the main group elements,^24^ L-edges of the third-row transition metals,^25^ and L-edges of the f block elements.^26^

Herein we report the reactivity of **1** with various oxidants that leads to the complete Sm(iii) halide series of **2-X** (X = F, Cl, Br, and I) where each member exhibits near trigonal planar geometry. This is the first molecular Sm series to be probed by fluorescence-detected Lα_1_ XAS at the Sm L_3_-edge where the resolved pre-edge feature is observed in each spectrum. The XAS data are subsequently interpreted with the aid of time-dependent density functional theoretical (TD-DFT) calculations. With a good reproduction of the observed experimental features that validates the theoretical model, the calculations disclose the salient factors that contribute to the X-ray spectrum which in turn provides insight into the electronic structure and bonding in these complexes.

## Experimental section

### General methods

The syntheses and manipulations described below were conducted under argon with rigorous exclusion of oxygen and water using a glove box, vacuum line, and Schlenk techniques. Hexane and toluene were purged with UHP grade argon (Airgas), passed through columns containing Q-5 and molecular sieves, stored over K mirrors and degassed before use. C_6_D_6_ (Cambridge Isotope Laboratories) was refluxed over K, degassed by three freeze–pump–thaw cycles, and vacuum-transferred before use. [Fe(Cp)_2_][PF_6_] and **1**^3^ were prepared according to literature procedures. The ^1^H (400 or 500 MHz), ^13^C{^1^H} (125 or 100 MHz), ^29^Si{^1^H} (99 or 80 MHz) and ^19^F (376 MHz) NMR spectra were obtained using a Bruker AV III HD 400 with a 5 mm BBO Prodigy probe or a Bruker AV III HD 500 with a 5 mm BBO Prodigy probe spectrometer and were referenced to SiMe_4_ (^1^H, ^13^C, and ^29^Si) or C_7_H_5_F_3_/CDCl_3_ (^19^F). Solution magnetic susceptibilities were determined by the Evans method.^27^ The FTIR samples were prepared as Nujol mulls in KBr discs using a PerkinElmer Spectrum RX1 spectrometer. Elemental analyses were performed either by using a PerkinElmer 2400 series II CHNS elemental analyser or with the assistance of Mrs. Anne Davies and Mr. Martin Jennings at The University of Manchester, UK.

### Synthesis of [Sm(N^††^)_2_(F)] (**2-F**)

[Sm(N^††^)_2_] (**1**, 1.62 g, 2.01 mmol) and [Fe(Cp)_2_][PF_6_] (0.665 g, 2.01 mmol) were cooled to –78 °C and toluene (30 mL) was added. The resultant red reaction mixture was allowed to warm to room temperature and stirred for 16 h. The mixture was concentrated *in vacuo* to *ca.* 5 mL, forming a precipitate, and was filtered. The supernatant was stored at –25 °C to give crystals of **2-F** and ferrocene. The mixture was heated to 100 °C *in vacuo* for 24 h to remove ferrocene by sublimation, leaving yellow crystals of **2-F** (0.66 g, 40%).

Anal. calcd for C_36_H_84_N_2_FSi_4_Sm: C, 52.30; H, 10.24; N, 3.39. Found: C, 51.09; H, 10.05; N, 3.03. Magnetic moment (Evans method, C_6_D_6_, 298 K): *μ*_eff_ = 1.65 *μ*_B_. ^1^H NMR (C_6_D_6_, 400 MHz): *δ* = –9.55 (br, *ν*_1/2_ ∼ 150 Hz, 12 H, C*H*(CH_3_)_2_), 0.55 (br, *ν*_1/2_ ∼ 15 Hz, 72 H, CH(C*H*_3_)_2_). ^13^C{^1^H} NMR (C_6_D_6_, 101 MHz): *δ* = 16.13 (s, *C*H(CH_3_)_2_), 19.81 (s, CH(*C*H_3_)_2_). ^29^Si{^1^H} NMR (C_6_D_6_, 99 MHz): *δ* = 13.57 (s). ^19^F NMR (C_6_D_6_, 376 MHz): not observed. IR (Nujol, cm^–1^): 1236 m, 1213 w, 1155 w, 1070 m, 1011 s, 995 s, 974 s, 945 s, 926 s, 880 s, 696 s, 660 s, 625 m.

### Synthesis of [Sm(N^††^)_2_(Cl)] (**2-Cl**)

A solution of [Sm(N^††^)_2_] (**1**, 4.04 g, 5.00 mmol) in toluene (30 mL) was cooled to –78 °C and treated dropwise with a ^*t*^BuCl (2.2 mL, 20 mmol) solution in toluene (10 mL). The dark green mixture was allowed to warm to room temperature and stirred for 3 h until a colour change to amber-yellow was observed. The volatiles were removed *in vacuo* to afford a yellow powder. The solids were extracted with hot hexane (40 mL) and concentrated to *ca.* 10 mL. Storage at –25 °C gave **2-Cl** as yellow crystals (3.59 g, 85%).

Anal. calcd for C_36_H_84_ClN_2_Si_4_Sm: C, 52.85; H, 10.35; N, 3.16. Found: C, 51.73; H, 10.17; N, 3.04. Magnetic moment (Evans method, C_6_D_6_, 298 K): *μ*_eff_ = 1.75 *μ*_B_. (C_6_D_6_, 400 MHz): *δ* = –9.65 (br, *ν*_1/2_ ∼ 200 Hz, 12 H, C*H*(CH_3_)_2_), 0.47 (br, *ν*_1/2_ ∼ 15 Hz, 72 H, CH(C*H*_3_)_2_). ^13^C{^1^H} NMR (C_6_D_6_, 126 MHz): *δ* = 15.62 (s, *C*H(CH_3_)_2_), 19.92 (s, CH(*C*H_3_)_2_). ^29^Si{^1^H} NMR (C_6_D_6_, 99 MHz): *δ* = 15.03 (s). IR (Nujol, cm^–1^): 1244 m, 1157 w, 1076 m, 1061 m, 1011 m, 991 m, 945 s, 880 s, 719 s, 694 s, 667 s, 636 s, 594 s.

### Synthesis of [Sm(N^††^)_2_(Br)] (**2-Br**)

A pre-cooled (–78 °C) solution of [Sm(N^††^)_2_] (**1**, 1.62 g, 2.01 mmol) in toluene (30 mL) as treated dropwise with a solution of ^*t*^BuBr (1.0 mL, 8.0 mmol) in toluene (10 mL). The dark green mixture was allowed to warm to room temperature and stirred for 40 min until a colour change to bright yellow was observed. The volatiles were removed *in vacuo* to afford a yellow powder. The solids were extracted with hot hexane (30 mL) and concentrated to *ca.* 10 mL. Storage at room temperature gave large orange crystals of **2-Br** (1.33 g, 75%).

Anal. calcd for C_36_H_84_BrN_2_Si_4_Sm: C, 48.71; H, 9.56; N, 3.16. Found: C, 48.65; H, 9.88; N, 3.13. Magnetic moment (Evans method, C_6_D_6_, 298 K): *μ*_eff_ = 1.68 *μ*_B_.^1^H NMR (C_6_D_6_, 500 MHz): *δ* = –9.14 (br, *ν*_1/2_ ∼ 200 Hz, 12 H, C*H*(CH_3_)_2_), 0.42 (br, *ν*_1/2_ ∼ 20 Hz, 72 H, CH(C*H*_3_)_2_). ^13^C{^1^H} NMR (C_6_D_6_, 126 MHz): *δ* = 15.56 (s, *C*H(CH_3_)_2_), 20.00 (s, CH(*C*H_3_)_2_). ^29^Si{^1^H} NMR (C_6_D_6_, 99 MHz): *δ* = 15.67 (s). IR (Nujol, cm^–1^): 1155 w, 1061 m, 1013 m, 993 m, 939 s, 880 m, 723 m, 694 s, 660 m, 617 m.

### Synthesis of [Sm(N^††^)_2_(I)] (**2-**I) and [{Sm(N^††^)}_3_(μ_2_-I)_3_(μ_3_-I)_2_] (**3**)

Iodine (0.898 g, 3.54 mmol) was dissolved in toluene (40 mL) and added dropwise to a pre-cooled (–78 °C) solution of [Sm(N^††^)_2_] (**1**, 5.78 g, 7.15 mmol) in toluene (25 mL). The solution was allowed to warm to room temperature and stirred for 5.5 h to give a yellow reaction mixture. The volatiles were removed *in vacuo* to afford a yellow powder. The solids were extracted with hot hexane (80 mL), filtered and concentrated to *ca.* 35 mL. Storage at –25 °C yielded yellow crystals of **2-I**, which were isolated and washed with pentane (3.53 g, 53%). Further concentration of the filtrate to *ca.* 15 mL and subsequent storage at –25 °C yielded brown crystals of **3** (0.36 g, 15%).

For **2-I**: Anal. calcd for C_36_H_84_IN_2_Si_4_Sm: C, 46.26; H, 9.06; N, 3.00. Found: C, 45.83; H, 9.09; N, 2.64. Magnetic moment (Evans method, C_6_D_6_, 298 K): *μ*_eff_ = 1.77 *μ*_B_. ^1^H NMR (C_6_D_6_, 400 MHz): *δ* = –8.47 (br, *ν*_1/2_ ∼ 200 Hz, 12 H, C*H*(CH_3_)_2_), 0.36 (br, *ν*_1/2_ ∼ 20 Hz, 72 H, CH(C*H*_3_)_2_). ^13^C{^1^H} NMR (C_6_D_6_, 101 MHz): *δ* = 15.38 (s, *C*H(CH_3_)_2_), 20.13 (s, CH(*C*H_3_)_2_). ^29^Si{^1^H} NMR (C_6_D_6_, 99 MHz): *δ* = 16.67 (s). IR (Nujol, cm^–1^): 1246 m, 1155 w, 1076 m, 1059 m, 1011 m, 989 m, 935 s, 883 m, 719 s, 696 s, 662 m, 637 s, 586 m.

For **3**: Anal. calcd for C_54_H_126_I_5_N_3_Si_6_Sm_3_·(C_6_H_14_)_0.5_: C, 32.37; H, 6.34; N, 1.99. Found: C, 32.53; H, 6.50; N, 1.99. Magnetic moment (Evans method, C_6_D_6_, 298 K): *μ*_eff_ = 3.77 *μ*_B_. ^1^H NMR (C_6_D_6_, 400 MHz): *δ* = –4.02 (br, *ν*_1/2_ ∼ 120 Hz, 6 H, C*H*(CH_3_)_2_), 0.40 (br, *ν*_1/2_ ∼ 20 Hz, 36 H, CH(C*H*_3_)_2_). ^13^C{^1^H} NMR (C_6_D_6_, 101 MHz): *δ* = 13.28 (s, *C*H(CH_3_)_2_), 23.80 (s, CH(*C*H_3_)_2_). ^29^Si{^1^H} NMR (C_6_D_6_, 400 MHz): not observed. IR (Nujol, cm^–1^): 1215 w, 1155 w, 1038 m, 916 m, 881 m, 708 s, 658 m, 633 m.

### X-ray spectroscopy

The Lα_1_ fluorescence-detected X-ray absorption spectra were collected at the C1 beamline of the Cornell High Energy Synchrotron Source (CHESS) under the ring conditions of 5 GeV and 125 mA. The incident X-rays were monochromated using a double Si(111) crystal monochromator. Internal energy calibrations were performed by simultaneous measurement of the Sm reference foil with the inflection point set to 6716.2 eV and all samples were calibrated with an equivalent shift. X-ray emission was monochromated using a set of five spherically bent Si crystals (422 reflection) maintained in a Rowland circle geometry with a Pilatus area detector.^28^ He-filled plastic bags were positioned in the beam flight path to minimize X-ray fluorescence attenuation. XAS was collected with a X-ray emission detector positioned at the Lα_1_ maximum for each complex (*ca.* 5636.1 eV). Data were collected from 6630 to 6850 eV. Step sizes of 10 eV, 0.2 eV, and 5 eV were used from 6630–6700 eV, 6700–6730 eV and 6730–6850 eV, respectively. The samples were maintained at 100 K during data collection through the use of a liquid N_2_ cryostream. The experimental spectra were averaged and normalised using PyMCA. For normalisation, the post-edge region (>6750 eV) was set to an absorbance of unity.

### Calculations

The program package ORCA was used for all calculations.^29^ Geometry optimisation employed the BP86 functional;^30^ single-point calculations on optimised and crystallographic coordinates with ^*i*^Pr groups truncated to Me used the PBE0 hybrid functional^31^ in conjunction with the RIJCOSX algorithm to expedite calculation of the Hartree–Fock exchange.^32^ The segmented all-electron relativistically contracted SARC-ZORA-TZVP basis set was used for samarium with an increased integration accuracy (SPECIALGRIDINTACC 10).^33^ The scalar relativistically recontracted ZORA-def2-TZVP basis set was used for all other atoms.^34^ The calculations included the zeroth-order regular approximation (ZORA) for relativistic effects^35^ as implemented by van Wüllen.^36^ The self-consistent field calculations were tightly converged (1 × 10^–8^*E*_h_ in energy, 1 × 10^–7^*E*_h_ in the charge density, and 1 × 10^–7^ in the maximum element of the DIIS^37^ error vector). The geometry was converged with the following convergence criteria: change in energy <10^–5^*E*_h_, average force <5 × 10^–4^*E*_h_ Bohr^–1^, and the maximum force 10^–4^*E*_h_ Bohr^–1^. The geometry search for all complexes was carried out in redundant internal coordinates without imposing geometric constraints. The Kohn–Sham canonical orbitals were obtained using Molekel.^38^

Time-dependent (TD-DFT) calculations of the samarium L_3_-edges were conducted as previously described for analogous K-pre-edge spectra.^16^,^39^–^42^ The TD-DFT calculations^43^ were performed allowing for only transitions from the samarium 2p orbitals^42^ which were localised using the Pipek-Mezey criteria.^44^ The TD-DFT equations were solved individually for each Sm 2p orbital,^41^ and the result a sum of the three subspectra. The absolute calculated transition energies are consistently underestimated because of the shortcomings in the ability of the DFT to model potentials near the nucleus. This results in the core orbitals being too high in energy relative to the valence, thus requiring a constant shift for a given absorber. It was established that constant shift of +78.6 eV for the Sm L_3_-edge was required for this level of theory. Plots were generated with a uniform Gaussian line broadening of 2.0 eV.

## Results and discussion

### Synthesis

**Scheme 1:**
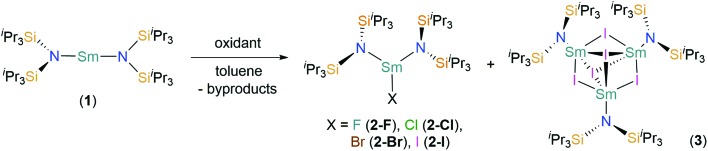
Synthesis of [Sm(N^††^)_2_(X)] (X = F, **2-F**; Cl, **2-Cl**; Br, **2-Br**; I, **2-I**) and [{Sm(N^††^)}_3_(μ_2_-I)_3_(μ_3_-I)_2_] (**3**) by the treatment of [Sm(N^††^)_2_] (**1**) with various oxidants; **2-F**: +[Fe(Cp)_2_][PF_6_], –[Fe(Cp)_2_], and PF_5_; **2-Cl**, **2-Br**: +^*t*^BuX, –CH_3_CH(CH_3_)CH_3_, and –CH_2_=C(CH_3_)CH_3_; **2-I**, **3**: +0.5 I_2_ and –unidentified by-products.

The near-linear Sm(ii) complex [Sm(N^††^)_2_] (**1**) was treated with various oxidants to give the heteroleptic Sm(iii) bis(silyl)amide halide complexes [Sm(N^††^)_2_(X)] (X = F, **2-F**; Cl, **2-Cl**; Br, **2-Br**; I, **2-I**) by single electron transfer (SET) (Scheme 1). On one occasion during the synthesis of **2-I** crystals of the trinuclear cluster [{Sm(N^††^)}_3_(μ_2_-I)_3_(μ_3_-I)_2_] (**3**) were isolated, presumably from the ligand redistribution (Schlenk-type equilibria) and subsequent aggregation. A four-fold excess of ^*t*^BuX (X = Cl and Br) was used to prepare **2-Cl** and **2-Br**, by SET from Sm(ii) to generate **2-X** and ^*t*^Bu˙ radicals; these combine to give the gaseous by-products 2-methylpropane and 2-methylpropene. This methodology is well-documented in the oxidation chemistry of Sm(ii); for example, [Sm(Cp*)_2_(Cl)(thf)] (Cp* = C_5_Me_5_) was first prepared by the reaction of [Sm(Cp*)_2_(thf)_2_] with ^*t*^BuCl.^45^ We found that **2-Cl** could not be synthesised from the direct reaction of SmCl_3_ with 2 eq. of KN^††^ in THF, where an intractable mixture of products was obtained. Presumably this occurs as a result of deprotonation of N^††^ coordinated to Sm(iii) by the second equivalent of KN^††^, and we have previously reported that bulky bis(silyl)amides readily form cyclometallates on Lewis acidic Ln centres in salt metathesis reactions,^4^ thus the oxidative route employed herein appears vital for the successful synthesis of **2-X**.

Although the yields of **2-Cl** (85%) and **2-B**r (75%) are relatively high, the analogous reagent ^*t*^BuF was considered unsuitable for the synthesis of **2-F** as the C–F bonds are far stronger than other C–X bonds.^46^ Thus, we utilised an alternative methodology: the synthesis of **2-F** was achieved in 40% yield by the oxidation of **1** with [Fe(Cp)_2_][PF_6_], with the concomitant elimination of ferrocene and PF_5_. A similar procedure had previously been employed for the synthesis of [Ce(N′′)_3_(F)] from [Ce(N′′)_3_] and [Fe(Cp)_2_][PF_6_].^47^ Whilst PF_5_ is released as a gas during the reaction, providing a thermodynamic driving force, ferrocene had to be removed *via* sublimation after the reaction was complete. Finally, **2-I** was synthesised by the direct treatment of **1** with 0.5 eq. of I_2_ in 53% yield, inspired by the synthesis of [Sm(Cp*)_2_(μ-I)]_3_ by sterically induced reduction.^48^ Although this reaction also produced a small quantity of the by-product **3**, this was easily separated by fractional recrystallisation, and thus we did not seek alternative reagents to optimise the synthesis of **2-I**. Low carbon values were consistently obtained for **2-F** and **2-Cl** during microanalysis experiments. We attribute this observation to carbide formation, which is a common occurrence for silicon-rich organometallic complexes, including those that contain N^††^,^6^,^7^ as all other analytical data were consistent with their bulk purity by comparison to **2-Br** and **2-I**.

Complexes **2-X** are remarkably robust towards ligand redistribution processes compared to similar heteroleptic unsolvated N′′ complexes, which is attributed to the steric demands of N^††^ precluding the formation of [Sm(N^††^)_3_]. It is noteworthy that full characterisation of the dimeric complexes [Ln(N′′)_2_(μ-Cl)]_2_ (Ln = Eu, Gd, and Yb) and monomeric [Y(N′′)_2_(Cl)(thf)_2_] was often hampered by the spontaneous formation of LnCl_3_ and [Ln(N′′)_3_],^49^ although the THF-solvated adducts [Ln(N′′)_2_(μ-X)(thf)]_2_ (X = Cl, Ln = Ce, Pr, Nd, Sm, and Dy; X = Br, Ln = Sm; X = I, Ln = La and Sm) appear more robust.^50^,^51^ Anwander and co-workers have recently shown that ligand redistribution processes are less prevalent when oxidative routes are applied to such species.^9^ Therefore, the increased steric bulk of N^††^ over N′′ allows for the preparation of three-coordinate monomeric heteroleptic complexes with no coordinated solvent, and this bulk also imparts the necessary kinetic stabilisation to circumvent ligand redistribution processes in such unsolvated complexes to allow **2-X** to be fully characterised.

### NMR spectroscopy

The ^1^H NMR spectra of **2-X** and **3** each exhibit two broad signals, corresponding to the methyl and methine protons of the ^*i*^Pr groups, which were assigned by relative integrations. Given that **3** formally contains one Sm(ii) and two Sm(iii) centres, we set the integrations based upon only the two Sm(iii) N^††^ groups being observed, making the assumption that the Sm(ii) N^††^ signals were not observed due to paramagnetic broadening. This phenomenon precludes the extraction of coupling constants in the ^1^H NMR spectra of all complexes; VT experiments were performed on all **2-X**, and despite the methine signals shifting and their linewidths reduced upon heating, these couplings could still not be resolved at 343 K (see the ESI†). Paramagnetically shifted methine resonances are attributed to close Sm···CH contacts in the solid state (*vide infra*) being maintained in solution due to the increased Fermi-contact term. The interactions in **2-X** are relatively strong as variable temperature measurements reveal these persist up to 343 K. Similarly strong Sm···CH contacts were previously seen for **1**,^3^ and are typical of low-coordinate f element silylamide complexes.^1^,^2^,^8^ Whilst the methyl signals of **2-X** and **3** were not paramagnetically shifted to any appreciable extent (all *δ*_H_ ∼ 0.3–0.6), the methine signals were exclusively found at high fields (*δ*_H_ = **2-F**, –9.55; **2-Cl**, –9.65, **2-Br**, –9.14; **2-I**, –8.47; **3**, –4.02 ppm). In contrast, we found that methyl and methine groups were observed in the ^13^C{^1^H} NMR spectra of **2-X** and **3** at similar chemical shifts to those seen previously for HN^††^,^6^ indicating that the paramagnetic shifting of these signals by Sm(iii) is relatively minor. The chemical shifts in the ^29^Si{^1^H} NMR spectra for **2-X** are more shielded for the most electronegative halides and follow a regular trend (*δ*_Si_ = **2-F**, 13.57; **2-Cl**, 15.03; **2-Br**, 15.67; **2-I**, 16.67 ppm), but no signals could be observed in the corresponding spectrum of **3**, presumably due to the larger paramagnetic effects of the formal Sm(ii) centre in this complex. No signal was observed in the ^19^F NMR spectrum of **2-F** as the fluoride is directly bonded to the Sm(iii) ion.

Room temperature magnetic moments for **2-X** and **3** (*μ*_eff_ = **2-F**, 1.65 *μ*_B_; **2-Cl**, 1.75 *μ*_B_; **2-Br**, 1.68 *μ*_B_; **2-I**, 1.77 *μ*_B_; **3**, 3.77 *μ*_B_) were determined by the Evans method.^27^ The values for **2-X** are consistent with each other considering weighing errors. Although a value of 0.85 *μ*_B_ is predicted for Sm(iii) with a ^6^H_5/2_ ground state, values between 1.4 and 1.8 *μ*_B_ are commonly observed at 298 K due to low-lying excited states mixing with the ground state.^52^ The magnetic moment of **3** is consistent with the values typically obtained for complexes containing a single Sm(ii) f^6^ centre at 298 K (3.3–3.5*μ*_B_), and thus we posit that the magnetic moments of the two Sm(iii) centres are opposed, with the net paramagnetism due to Sm(ii) only.

### Structural characterisation

**Fig. 1:**
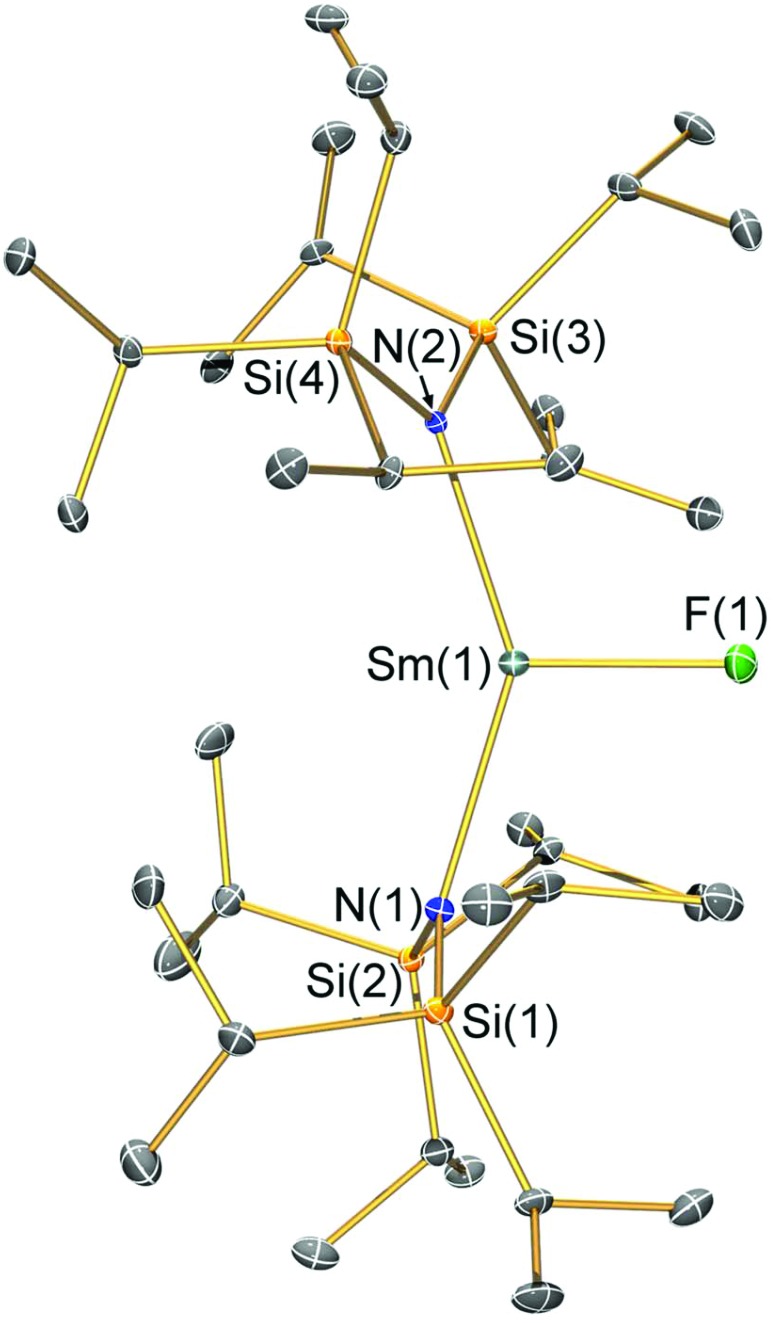
Molecular structure of [Sm(N^††^)_2_(F)] (**2-F**) with selective atom labelling. Displacement ellipsoids set at a 30% probability level and hydrogen atoms are omitted for clarity. Selected bond lengths [Å]: Sm(1)–F(1) 2.051(3), Sm(1)–N(1) 2.353(3), and Sm(1)–N(2) 2.334(3). Selected bond angles [°]: N(1)–Sm(1)–N(2) 143.29(12), N(1)–Sm(1)–F(1) 106.81(12), and N(2)–Sm(1)–F(1) 108.63(12).

**Fig. 2:**
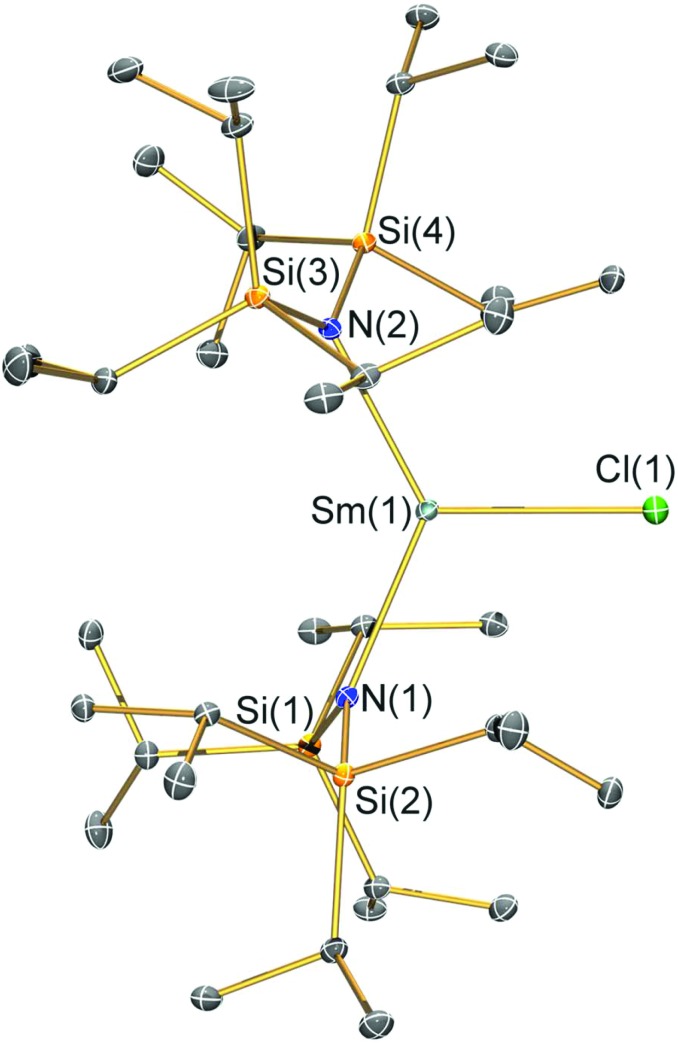
Molecular structure of [Sm(N^††^)_2_(Cl)] (**2-Cl**) with selective atom labelling. Displacement ellipsoids set at a 30% probability level and hydrogen atoms are omitted for clarity. Selected bond lengths [Å]: Sm(1)–Cl(1) 2.5813(7), Sm(1)–N(1) 2.295(2), and Sm(1)–N(2) 2.317(2). Selected bond angles [°]: N(1)–Sm(1)–N(2) 128.24(7), N(1)–Sm(1)–Cl(1) 111.34(6), and N(2)–Sm(1)–Cl(1) 117.11(5).

**Fig. 3:**
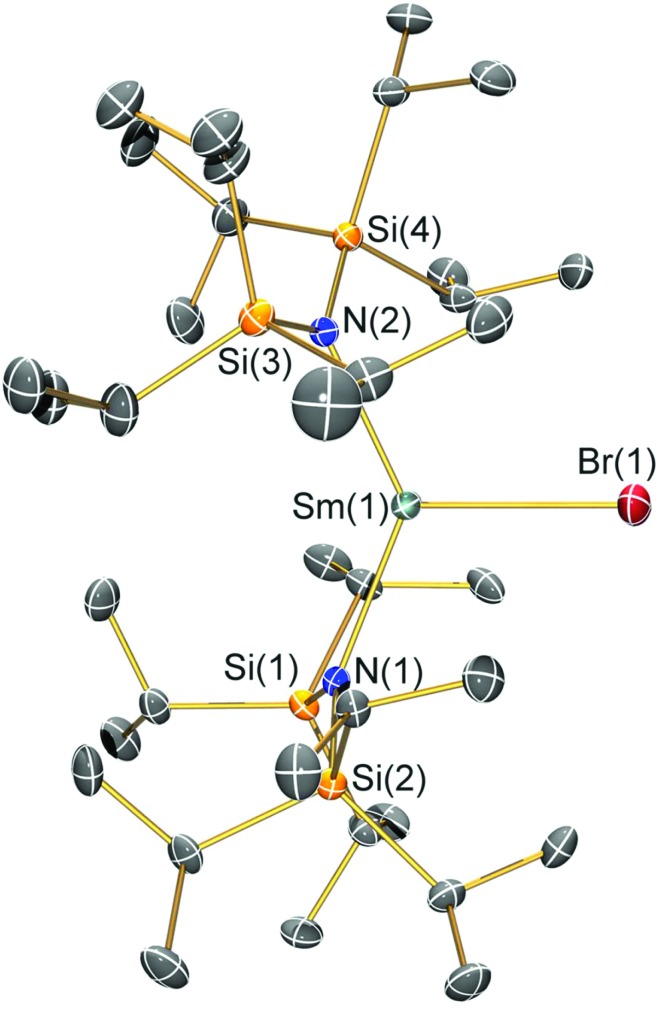
Molecular structure of [Sm(N^††^)_2_(Br)] (**2-Br**) with selective atom labelling. Displacement ellipsoids set at a 30% probability level and hydrogen atoms are omitted for clarity. Selected bond lengths [Å]: Sm(1)–Br(1) 2.7429(7), Sm(1)–N(1) 2.308(4), and Sm(1)–N(2) 2.324(4). Selected bond angles [°]: N(1)–Sm(1)–N(2) 133.52(14), N(1)–Sm(1)–Br(1) 112.19(10), and N(2)–Sm(1)–Br(1) 113.19(10).

**Fig. 4:**
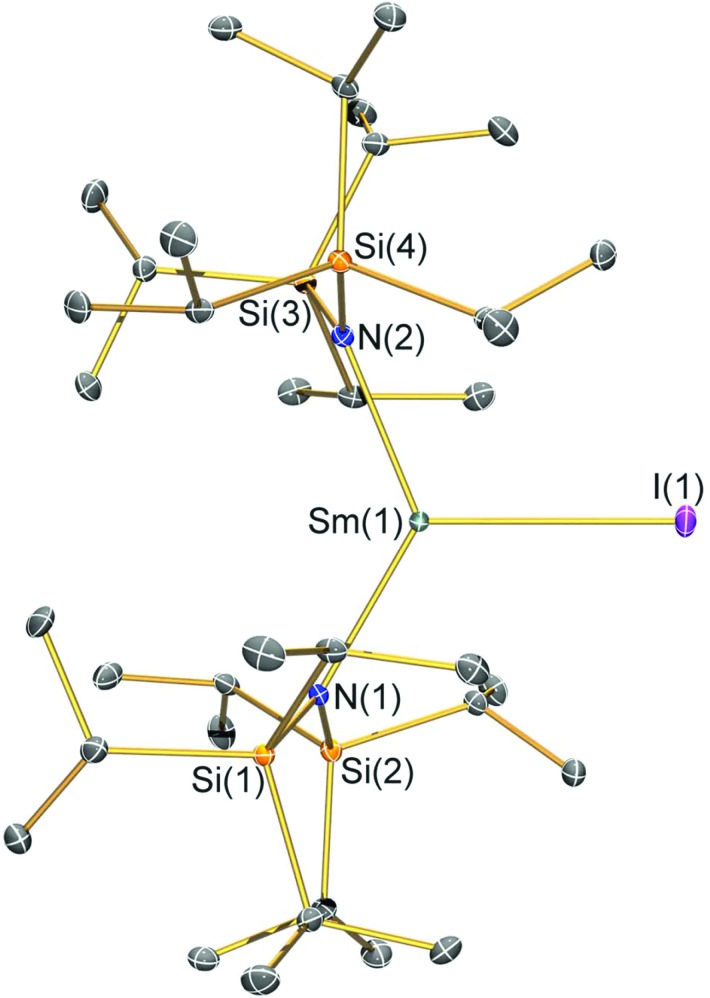
Molecular structure of [Sm(N^††^)_2_(I)] (**2-I**) with selective atom labelling. Displacement ellipsoids set at a 30% probability level and hydrogen atoms are omitted for clarity. Selected bond lengths [Å]: Sm(1)–I(1) 3.0199(7), Sm(1)–N(1) 2.323(3), and Sm(1)–N(2) 2.289(3). Selected bond angles [°]: N(1)–Sm(1)–N(2) 128.07(10), N(1)–Sm(1)–I(1) 119.75(7), and N(2)–Sm(1)–I(1) 112.16(7).

Single crystal X-ray diffractometry was performed to determine the solid-state structures of **2-X** and **3** (Fig. 1–4 and Table 1, see the ESI† for the structure of **3**). Interestingly, although **2-X** all crystallised in different space groups they are structurally analogous. Thus, we discuss them together. Complexes **2-X** are all monomeric in the solid state, with two monodentate N^††^ ligands and one halide giving distorted trigonal planar geometries with the Sm(iii) centres slightly out of the equatorial plane defined by one X and two N atoms to varying extents (Table 1). Whilst the N–Sm–N and N–Sm–X angles deviate significantly from an ideal 120° for all **2-X** due to the steric demands of N^††^, this is more pronounced for **2-F** than for other members of the series due to the small size of F (Table 1). The Sm–X distances in **2-X** increase regularly with the halide size (six-coordinate ionic radii: F^–^, 1.33 Å; Cl^–^, 1.88 Å; Br^–^, 1.96 Å; I^–^, 2.20 Å).^53^ The N′′ analogues [Sm(N′′)_2_(μ-X)(thf)]_2_ (X = Cl and Br)^8^,^51^ are five-coordinate and dimeric in the solid state, and as expected the mean bridging Sm–Cl [2.782(3) Å] and Sm–Br [2.955(2) Å] distances in these complexes are longer than the corresponding terminal distances in **2-Cl** and **2-Br**. However, the mean Sm–N distances in [Sm(N′′)_2_(μ-X)(thf)]_2_ [X = Cl, 2.27(1) Å; Br, 2.26(1) Å]^8^,^51^ are shorter than those in **2-Cl** and **2-Br**, highlighting the increased steric bulk of N^††^ over N′′. The mean Sm–N distances are *ca.* 0.03 Å longer for **2-F** than for the rest of the series, but all distances are much shorter than that seen for **1** [Sm–N_avg_: 2.483(8) Å],^3^ as expected for the smaller Sm(iii) ion (seven-coordinate ionic radii: Sm^3+^, 1.02 Å; Sm^2+^, 1.22 Å).^53^ As the N–Sm–N angles in **2-X** do not decrease regularly with the halide size we conclude that crystal packing effects have a significant influence on geometries. The bulk features of **2-I** are comparable with the recently reported U(iii) homologue, [U(N^††^)_2_(I)],^54^ but the structures of **2-X** are best compared with [Sm(N^††^)_2_(TEMPO)], which exhibits longer Sm–N distances [2.374(4) Å] and a smaller N–Sm–N angle [125.07(9)°] due to the larger size of TEMPO,^5^ and [Sm(N′′)_2_(I)(thf)_2_], which has a longer Sm–I distance [3.1011(2) Å] as this solvated complex is five-coordinate.^8^ The coordination spheres of the Lewis acidic Sm(iii) centres in **2-X** are completed by a number of short electrostatic Sm···CH contacts with both the methine and methyl fragments of the ^*i*^Pr groups, which according to variable temperature ^1^H NMR spectroscopy persist in solution up to 343 K for **2-X** (*vide supra*).

**Table 1:** Selected crystallographic distances (Å) and angles (°) for **2-X**

	**2-F**	**2-Cl**	**2-Br**	**2-I**
Sm–X	2.051(3)	2.5813(7)	2.7429(7)	3.0199(7)
Sm–N_avg_	2.344(4)	2.306(3)	2.316(6)	2.306(4)
N–Sm–N	143.3(1)	128.24(7)	133.5(1)	128.1(1)
N–Sm–X_avg_	107.7(2)	114.23(8)	112.7(8)	116.0(1)
Sm···N_2_X_plane_	0.141(2)	0.250(2)	0.145(2)	0.015(2)

The solid-state structure of **3** contains three independent molecules with similar geometrical parameters (Fig. S1†), and thus we only include the data for one of these clusters in this discussion for brevity. The Sm_3_I_5_ cluster in **3** contains three Sm centres that are bridged by three μ_2_-iodo ligands to form a Sm_3_I_3_ ring, which is capped with two μ_3_-iodides. An N^††^ ligand completes the coordination sphere of each 5-coordinate Sm centre. As complex **3** possesses eight monoanionic ligands it formally contains one Sm(ii) and two Sm(iii) centres, and so can be formally described as an aggregate of a Sm(ii) complex [Sm(N^††^)(I)] with the dinuclear Sm(iii) complex [Sm(N^††^)(I)_2_]_2_. Due to the poor quality of the diffraction data we cannot draw further conclusions about the structure of **3** from bond lengths and angles, however, the identity is unambiguous and in line with other characterisation.

### Electronic spectroscopy

**Fig. 5:**
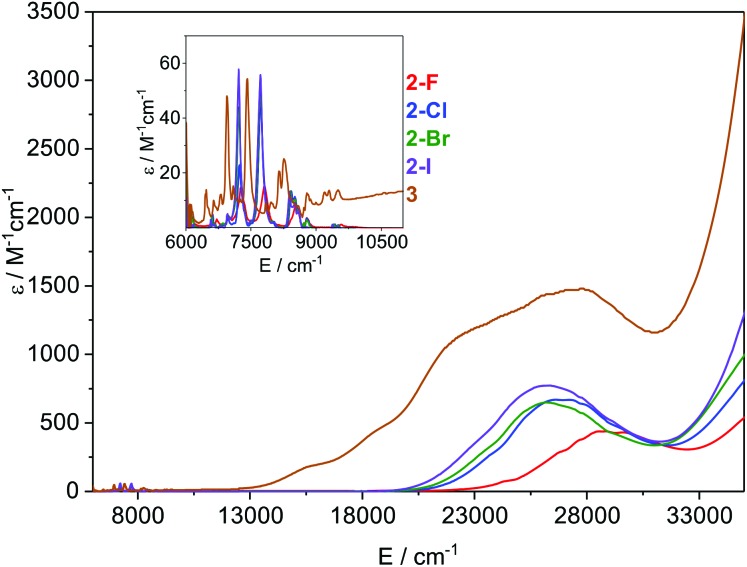
Overlay of the electronic spectra of **2-X** and **3** in toluene at ambient temperature. The inset shows the relative absorbance in the NIR region.

The electronic absorption spectra of **2-X** and **3** recorded in toluene at ambient temperature are presented in Fig. 5. All visible spectra are dominated by charge transfer (CT) bands tailing in from the UV region; it is noteworthy that for **2-Cl**, **2-Br** and **2-I** these absorptions are similar (*λ*_max_ ∼ 370–380 nm; *ν̃* ∼ 26 500 cm^–1^, and *ε* ∼ 650–750 M^–1^ cm^–1^), with the intensities increasing with the halogen size. For **2-F** this feature is blue-shifted and is considerably less intense than for the other three complexes (*λ*_max_ ∼ 350 nm, *ν̃* ∼ 28 500 cm^–1^, and *ε* ∼ 430 M^–1^ cm^–1^). The corresponding CT band for **3** (*λ*_max_ ∼ 365 nm, *ν̃* ∼ 27 500 cm^–1^, and *ε* ∼ 1500 M^–1^ cm^–1^) is around twice the intensity of that observed for **2-Cl** and covers most of the visible region. It is noteworthy that this band contains three shoulders at lower energy, which could arise from the f–d transitions owing to the formal presence of a Sm(ii) ion in this complex. In the NIR region, relatively intense f–f absorptions are observed for all complexes (Fig. 5 inset); again for **2-F** these signals are slightly blue-shifted compared to the other three halides. We assign these absorptions to the series of ^6^H_5/2_ → ^6^F_11/2,9/2,7/2,5/2,1/2_ transitions; the three most intense peaks are seen at *ν̃* ∼ 7200, 7800, and 8500 cm^–1^ for **2-X** (Fig. S30–S33†). Again the intensities of these peaks increase with the halogen size and this is most clearly seen for the absorption at *ν̃* ∼ 7200 cm^–1^, where the ε values range from ∼15 M^–1^ cm^–1^ for **2-F** to 55 M^–1^ cm^–1^ for **2-I**. The spectrum of **3** contains comparable absorptions in the NIR region, with a clear bathochromic shift of ∼500 cm^–1^ for the three most intense absorptions. This arises from the markedly different ligand field of this complex compared to **2-X**.

### X-ray spectroscopy

**Fig. 6:**
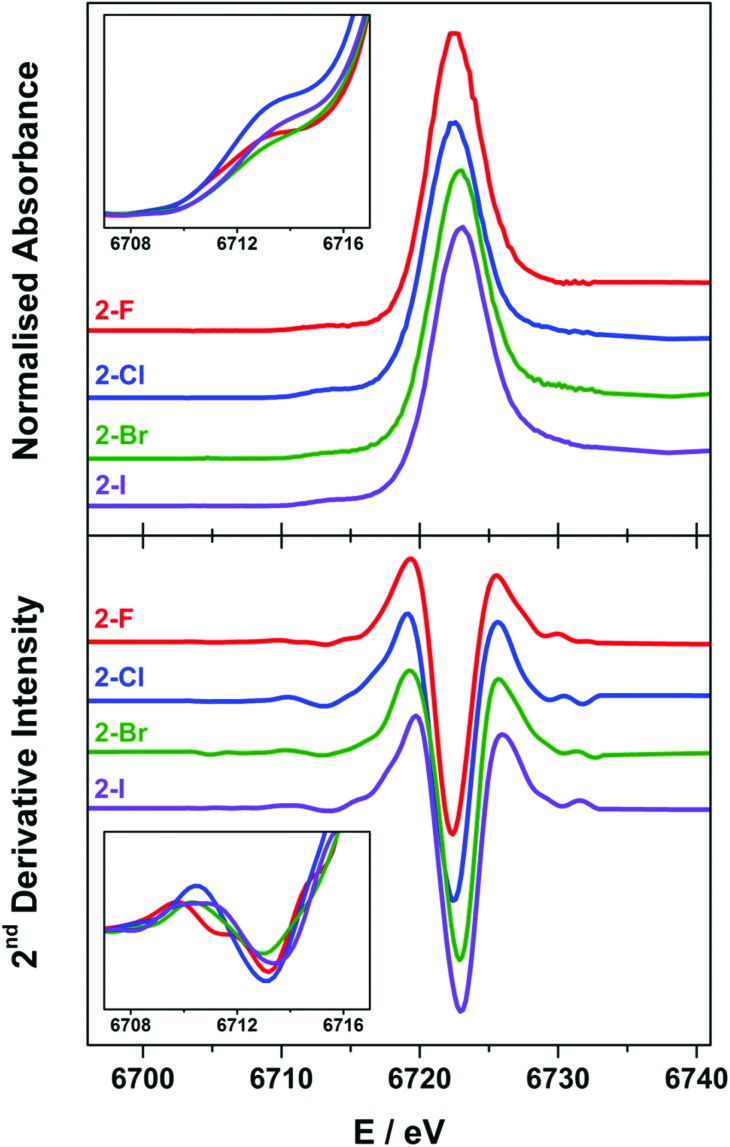
Comparison of the normalised Sm L_3_-edge XAS (top) and their FFT-smoothed second derivative spectra (bottom) of the **2-X** (X = F, Cl, Br, and I) series recorded at 100 K. The insets show the expansion of the overlaid FFT-smoothed pre-edge peaks (top) and their second derivatives (bottom).

The Lα_1_-detected X-ray absorption spectra recorded at the Sm L_3_-edge are dominated by a prominent absorption peak at *ca.* 6720 eV referred to as the white-line,^11^,^55^ identified as the 2p → 6s and 2p → 5d electronic transitions, which are both dipole-allowed. Specifically, the L_3_-edge represents excitation from 2p_3/2_ to both 5d_3/2_ and 5d_5/2_ states, and is preferred to the L_2_-edge which is limited to transitions from the 2p_1/2_ to the 5d_3/2_ state and therefore half as intense.^56^,^57^ For Ln ions whose valence electrons reside in the contracted 4f subshell, the dipole forbidden but quadrupole-allowed 2p → 4f electronic transitions constitute the pre-edge region at the foot of the white-line peak.^56^ The Sm Lα_1_ fluorescence-detected spectra of the **2-X** series are presented in Fig. 6. Each spectrum has been normalised to the step in the continuum across the absorption edge, and the pre-edge and white-line energies and intensities are listed in Table 2.

**Table 2:** Experimental and calculated^*a*^ Sm L_3_-edge XAS data

	Pre-edge^*b*^	Intensity^*c*^	White-line^*b*^	Intensity^*d*^
**2-F**	6713.0	0.09	6722.5	18.9
	(6717.3)		(6723.2)	
**2-Cl**	6713.2	0.13	6722.5	14.7
	(6717.2)		(6722.7)	
**2-Br**	6713.1	0.08	6722.9	13.6
	(6717.3)		(6722.6)	
**2-I**	6713.4	0.11	6723.1	16.5
	(6717.3)		(6722.2)	

^*a*^Calculated values in parenthesis are shifted +78.6 eV.

^*b*^Energy minimum in the second derivative spectrum.

^*c*^Pre-edge peak height, in arbitrary units.

^*d*^Area under the single Gaussian fit to the white-line peak after the subtraction of the edge, in arbitrary units.

The white-line energies across the **2-X** series are almost invariant, falling into the range 6722.8 ± 0.3 eV, with a slight shift to higher energy with the increasing halide size though within the experimental error.^17^,^58^ These energies are identical to the Sm L_3_-edge energies recorded for the related charge-neutral, three-coordinate Sm(iii) compounds, including [Sm(C_5_H_4_SiMe_3_)_3_] at 6723.2 eV and [Sm(N′′)_3_] at 6722.8 eV.^14^ The most noticeable difference across the series is the intensity of the white-line, based on the fit of a single Gaussian to the peak after the removal of the ionisation edge. The intensity across the series trends **2-F** > **2-I** > **2-Cl** > **2-Br**, and therefore is not an electronic effect of the halide ligand. The spectra of **2-Cl** and **2-Br** have a more prominent shoulder to higher energy beyond the white-line peak which is not wholly included in the estimate of the peak intensity. This stems from the complex geometry,^59^ which varies across the series because of the electronic and steric factors associated with each halide ligand. In addition, there are scattering contributions that also trend with the increasing atomic number of the halide.^60^ Complexes **2-Cl** and **2-Br** exhibit a more skewed L_3_-edge profile and also show the largest deviation from a trigonal planar geometry about the Sm(iii) ion (Table 1). The longer Sm–X bonds in **2-Cl**, **2-Br** and **2-I** will ensure that the crystal packing effects will generate a larger distribution of geometric distortions than for the more compact **2-F** species. This will modify the energetic splitting of the 5d orbitals, broadening the white-line peak.

The enhanced resolution of the Lα_1_ fluorescence spectrum yielded a resolved pre-edge feature in the **2-X** series (Fig. 6). As with the white-line peak, the pre-edge peak increases in energy with the increasing size of the halide,^17^,^58^ though for the **2-X** series it only spans the narrow range at 6713.2 ± 0.2 eV, residing 9.3–9.8 eV below the white-line (Table 2). This matches the pre-edge energy observed from the high-resolution X-ray spectral studies performed on Ce_2_(CO_3_)_3_ where the pre-edge precedes the white-line by 8.5 eV, to Dy(NO_3_)_3_ and Yb_2_O_3_ where the separation increases to 10.2 eV.^21^,^61^ The intensity of the pre-edge peak was estimated from the height of the spectral feature, with **2-Cl** the most intense and **2-Br** the least (Fig. 6 inset). It should be noted that the pre-edge peak coincidentally matches the white-line energy for Sm(ii) (*vide infra*) and therefore could plausibly arise from the photoreduction of the Sm(iii) compound during measurement. However, the variation in the pre-edge intensity does not follow the expected trend in reduction potentials where **2-I** is the easiest to reduce and **2-F** the most difficult. Moreover, the rate of decomposition is too slow under these experimental conditions to have appreciable amounts of the reduced Sm(ii) species generated during the measurement.^14^

The Sm L_3_-edge spectrum of **1**, which possesses a two-coordinate Sm(ii) ion gave two prominent features (Fig. S35†). Both are white-line peaks, with the lower energy feature at 6709.6 eV assigned to the Sm(ii) f^6^ centre. The higher peak at 6718.8 eV stems from the oxidation of the highly air-sensitive complex during sample preparation. Despite our best attempts in excluding air and solvents in the sample preparation, we were unsuccessful in obtaining a spectrum exclusively of the Sm(ii) centre in **1**. It should be noted that other investigators have also encountered difficulties in measuring the spectra of low-valent, low-coordinate Ln complexes.^14^ This energy matches the white-line peak for the Sm(iii) ions in the **2-X** series, and shows a shift of 9.2 eV upon one-electron reduction of a Sm(iii) to Sm(ii) as noted in other L_3_-edge XAS studies. This difference is noticeably larger than the energy separation of 7.6 eV for three-coordinate [Sm^II^(C_5_H_4_SiMe_3_)_3_]^1–^ and [Sm^III^(C_5_H_4_SiMe_3_)_3_],^14^ and the 6.0–7.5 eV separation of the white lines of Sm(ii) and Sm(iii) ions at octahedral sites in mixed-valence solids.^12^,^62^ This highlights the effect of coordination number on the ionisation energy, and therein the stability and reactivity of **1**.

In the current study of low-coordinate Sm(iii) complexes, it was advantageous to utilise a simple TD- DFT method to reproduce the experimental X-ray absorption spectra.^41^,^42^ Such an approach has proven successful in calculating the metal and ligand absorption edges in a variety of complexes possessing first-row transition metals,^22^,^39^,^63^,^64^ sulfur-donor ligands,^16^,^39^,^40^,^64^,^65^ chloride ligands,^41^,^66^ and most recently lanthanide complexes.^14^

**Fig. 7:**
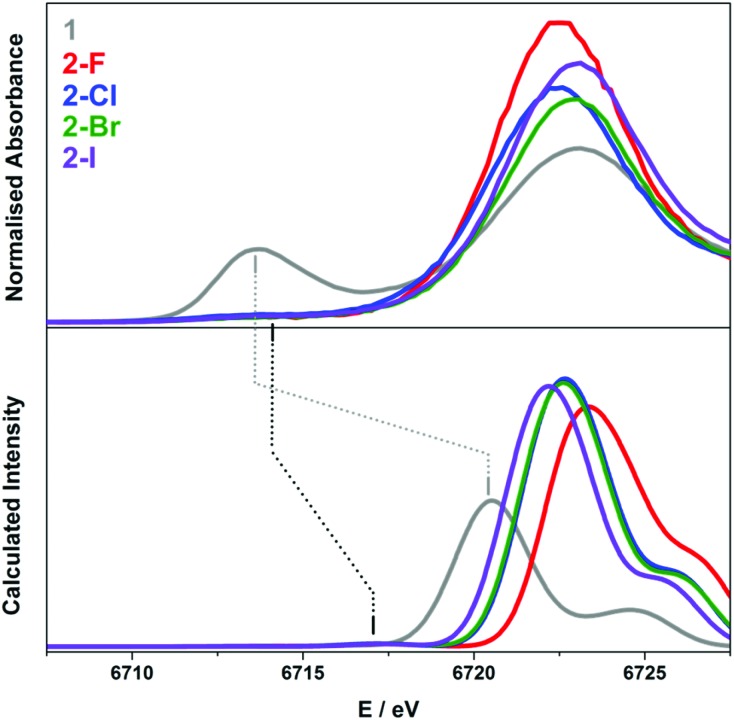
Comparison of the experimental (top) and calculated (bottom) Sm L_3_-edge spectra of **1** (grey), **2-F** (red), **2-Cl** (blue), **2-Br** (green) and **2-I** (violet) obtained from the ZORA-PBE0 TD-DFT calculations. The calculated spectra are shifted +78.6 eV with the intensity in arbitrary units. The grey dashed line links the experimental and calculated Sm(ii) white-line peaks; the black dashed line links the experimental and calculated Sm(iii)–X pre-edge peaks.

The calculation of the L_3_-edge spectra was conducted on the crystallographic coordinates for each member of the **2-X** series employing a large segmented all-electron relativistically contracted (SARC) basis set for samarium and the PBE0 hybrid functional. Due to the limitations in the accurate treatment of the excited states in DFT, the *absolute* transition energies cannot be obtained by this method. Nevertheless, the *relative* transition energies and the *relative* intensities are, in general, reliably modelled. For a given theoretical method, that is, a combination of functional, basis sets, relativistic treatment, and so forth, an empirical correction of +78.6 eV is applied to the calculated Sm L_3_-edge spectra in order to align with the experimental data as shown in Fig. 7.

A reasonable fit is afforded by the calculated Sm L_3_-edge spectra, in particular the relative intensity of the dominant white-line and pre-edge features. The data were shifted so as to align with the white-line energy despite this feature derived from the virtual (unoccupied) 5d and 6s rbitals of Sm whose energies represent a challenge for DFT to accurately compute since it stems from a ground state electronic structure. The computed white-line shifts to higher energy with the decreasing atomic number of the halide such that **2-I** is 1 eV higher in energy than **2-F** (Fig. 7). The white-line energy, which is an estimate of the dipole-allowed 2p → 5d/6s transition, is dependent on the ligand field as the bis(silyl)amide and halide ligands will form bonding interactions with these frontier orbitals. As shown in Fig. S36† where the TD-DFT derived Sm L_3_-edge spectra for the crystallographic and geometry-optimised structures of **2-Cl** are compared, the white-line shifts to slightly higher energy as a function of the Sm–Cl bond distance, which is 2.5813(7) Å in the solid-state structure compared to 2.662 Å in the geometry-optimised one (Table S2†). As a result, the 5d orbitals are destabilised in the former shifting its white-line 0.7 eV higher in energy. It is likely that the variation in the Sm–X bond length of the larger halides (Cl, Br, and I) leads to a broadening of the dominant 2p → 5d/6s peak compared to that of **2-F** where the more compact and tightly bound F^–^ ion deviates little from its crystallographic distance of 2.051(3) Å (Table 1). It appears that the Sm–X distance provides the greatest contribution to the L_3_-edge profile, with deviations from the planarity and changes to the Sm–N bond lengths and angles having a less of an impact.

The calculated pre-edge peaks for **2-X** are all identical within the experimental error at 6717.3 V (Table 2). Most noticeably is the DFT underestimation of the degree of 4f contraction in these Sm(iii) complexes, *i.e.* the separation of the 4f and 5d subshells. The TD-DFT computed pre-edge is *ca.* 4 eV shy of the experimental separation, underscoring the limitations of DFT in the defining energies of the virtual states, in this case the 5d and 6s orbitals to which the calculated spectra are aligned. Moreover, TD-DFT neglects a spin–orbit treatment of the excited states arising from the 2p^5^ final electron configuration. Nevertheless, the approach is sufficient to carry out a semi-qualitative interpretation of the Sm L_3_-edge data. The pre-edge region comprises two sets of transitions, namely the excitation of a spin-up electron to the unoccupied f_*z*^3^_ and f_*y*(*y*^2^–3*x*^2^)_ orbitals in the α-spin manifold because these f orbitals have probability density projected toward the halide and silylamide ligands in this trigonal ligand field (Fig. S37†). The two transitions are the lowest energy features of the pre-edge. The higher energy transitions are the excitation of a spin-down electron to all seven unoccupied β-spin f orbitals of the Sm(iii) f^5^ ion. As is often the case, TD-DFT overestimates the polarisation of the valence orbitals, which is the stabilisation of the α-spin manifold compared to the β-spin manifold inherent to paramagnetic centres (Sm(iii) has five unpaired (spin-up) electrons that give the ^6^H_5/2_ ground state). The separation of the α-spin and the β-spin excitation is uniform across the **2-X** series (Fig. S37†). Overall, the relative intensity of the pre-edge peak is well-reproduced, and ascribed to a lack of s and d content to the contracted f orbitals such that the pre-edge transitions are purely quadrupole-allowed.^56^,^57^

The calculated L_3_-edge for **1** is significantly underestimated by this regime of hybrid functional and basis set. After applying the constant shift of +78.6 eV to the calculated data, the white-line is 2.4 eV lower than the computed L_3_-edge of the Sm(iii) complexes (Fig. 7). This is in contrast to the 9.2 eV separation observed experimentally. This shortcoming of the theory is related to the choice of the functional, as demonstrated recently by Fieser *et al.* who revealed that calculations with the GGA (generalised gradient approximation) functionals PBE and BLYP gave a similar 2–3 eV difference in white-line energies for Sm(ii) and Sm(iii) complexes with the best match obtained with the BHandHLYP hybrid functional with 50% Hartree–Fock exchange.^14^

## Conclusion

The aforementioned Sm–X series comprising four structurally homologous compounds where X = F, Cl, Br, and I, has been examined using Sm L_3_-edge XAS producing spectra with resolved pre-edge and white-line features. The straightforward TD-DFT protocol used previously for K-pre-edge spectra has been successfully used to reproduce the key spectral features, and subsequent examination revealed the pre-edge peak to comprise excitation from the 2p level to the acceptor orbitals with a wholly 4f character. It is perhaps not surprising that the contracted f orbital manifold is effectively unperturbed (within the resolution limits of L-edge XAS) by the coordination environment considering the weak donor strength of halide ligands. On the other hand, the dominant L_3_ white-line peak shows greater sensitivity to the ligand field. The calculations reveal that the peak position and width are governed by Sm–X bond lengths. Complex **2-F** has the narrowest white-line peak as the F^–^ ion is held close to the Sm(iii) ion ensuring that it remains unaffected by crystal packing. At the other end of the scale, the position and bulk of the I^–^ ligand leads to the broadening of the white-line peak due to small structural distortions of the geometry about the Sm(iii) ion from intermolecular interactions. The calculated white-line shifts to higher energy with the decreasing atomic number of the halide which reveals that the interaction of the halide is essentially electrostatic in perturbing the energy of the Sm 5d orbitals. The good correlation between the experiment and the theory highlights the utility of fluorescence-detected L-edge XAS as a valuable probe to explore the electronic structure and bonding in complexes across the whole f block.

## Conflicts of interest

There are no conflicts to declare.

## Supplementary Material

Supplementary information

Crystal structure data
