# Supplementary material for: Heteroleptic samarium(iii) halide complexes probed by fluorescence-detected L3-edge X-ray absorption spectroscopy
Source: Dalton Trans. 2018 May 4;47(31):10613–25. doi: 10.1039/c8dt01452c (PMC6083822; doi:10.1039/c8dt01452c)
Supplement: Supplementary file 1 [file DT-047-C8DT01452C-s001.pdf]

Electronic Supplementary Material (ESI) for Dalton Transactions.
This journal is © The Royal Society of Chemistry 2018

Electronic Supplementary Information

Heteroleptic Samarium(III) Halide Complexes Probed by Fluorescence-Detected L_3-Edge X-ray Absorption Spectroscopy

Conrad A. P. Goodwin,[a] *Benjamin L. L. Réant,*[a] *Jon G. C. Kragshow,*[a] *Ida M. DiMucci,*[b] *Kyle M. Lancaster,**[b] *David P. Mills**[a] *and Stephen Sproules**[c]

[a] School of Chemistry, The University of Manchester, Oxford Road, Manchester M13 9PL, UK

[b] Department of Chemistry and Chemical Biology, Baker Laboratory, Cornell University, Ithaca, New York 14853, USA

[c] WestCHEM, School of Chemistry, University of Glasgow, Glasgow G12 8QQ, UK

E-mail: kml236@cornell.edu; david.mills@manchester.ac.uk; stephen.sproules@glasgow.ac.uk

Contents

1. Molecular structure of **3** 2
2. NMR Spectroscopy 3
3. FTIR Spectroscopy 14
4. Electronic Spectroscopy 17
5. Crystallography 20
6. X-ray Absorption Spectroscopy 22
7. References 36

1. Molecular structure of 3

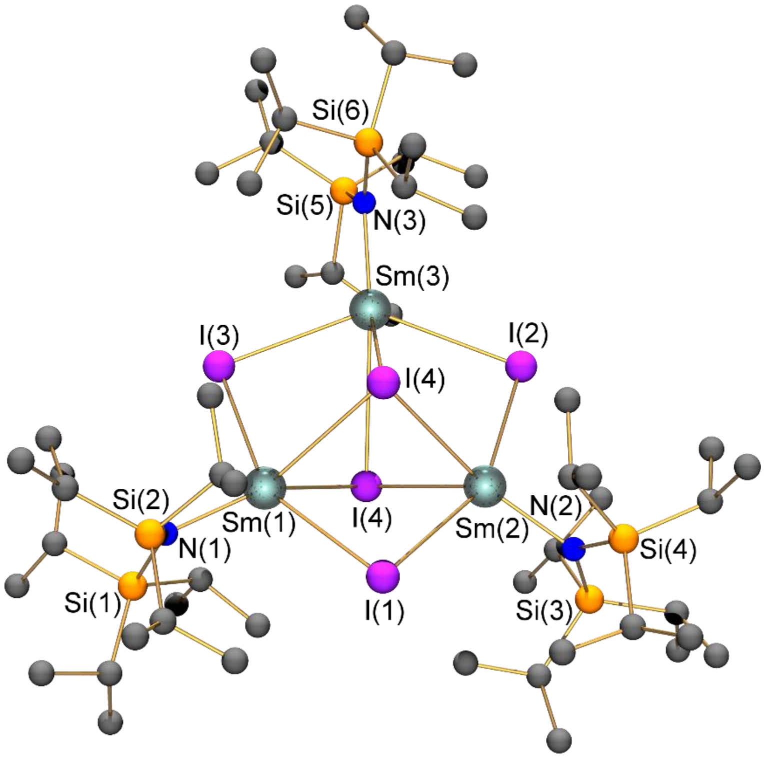

Fig. S1 Ball and stick rendering of the molecular structure of one of the clusters in **3**. The data quality does not permit meaningful analysis of bond metrics.

2. NMR Spectroscopy

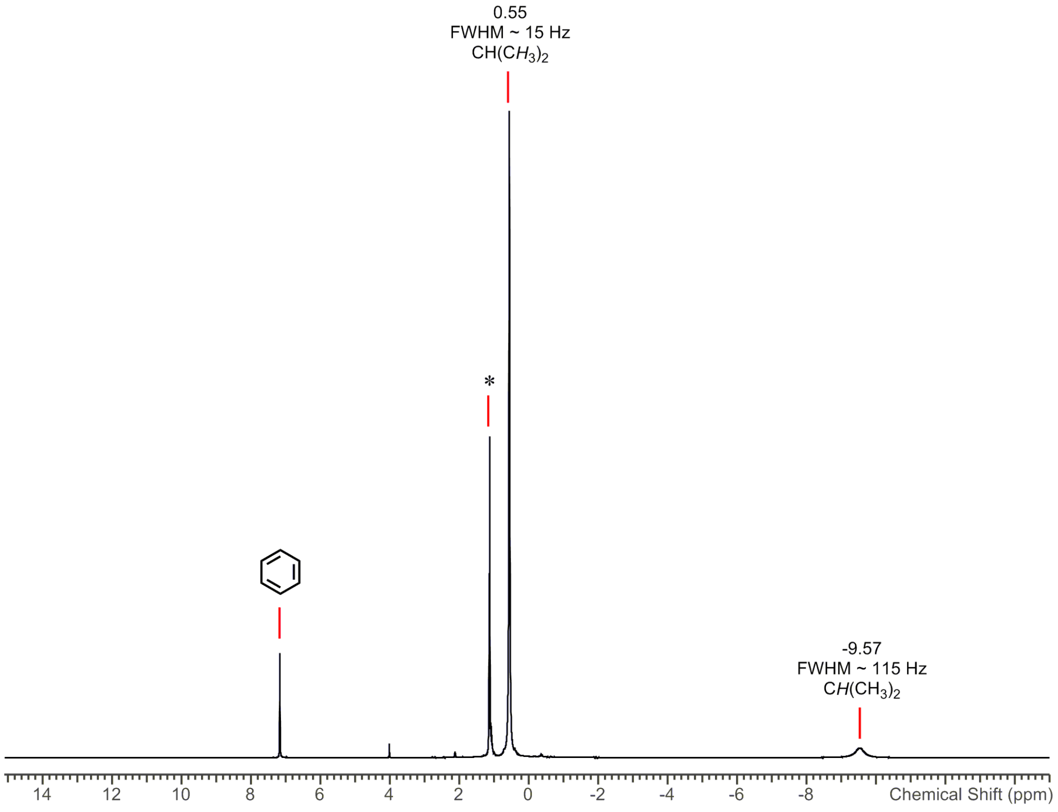

Fig. S2 ^{1}H NMR spectrum of **2-F** in C_6D_6 (* denotes $HN(Si^iPr_3)_2$)

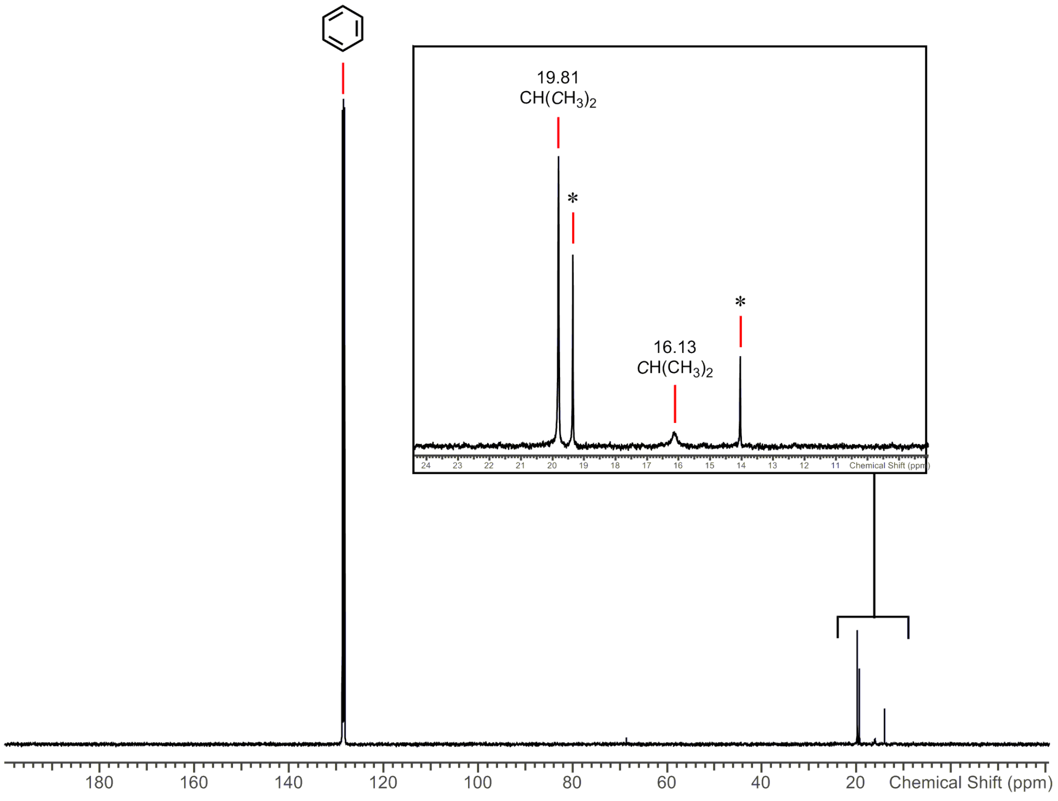

Fig. S3 $^{13}C\{^1H\}$ NMR spectrum of **2-F** in C_6D_6 (* denotes $HN(Si^iPr_3)_2$)

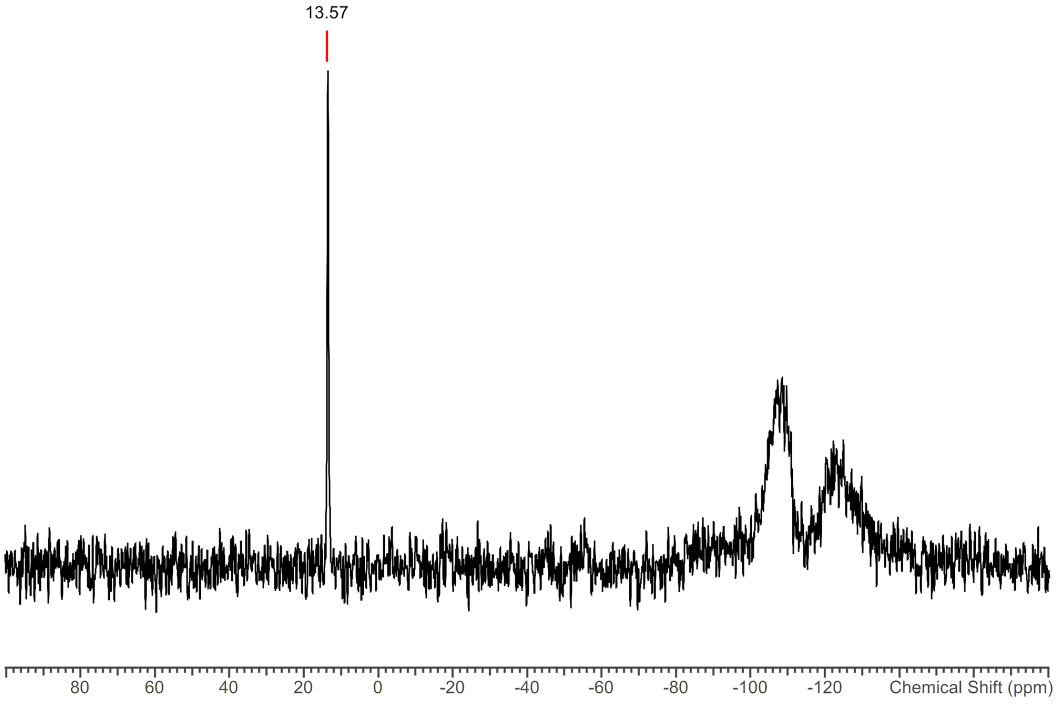

Fig. S4 $^{29}Si\{^1H\}$ NMR spectrum of **2-F** in C_6D_6. Broad features between -100 to -120 are glass.

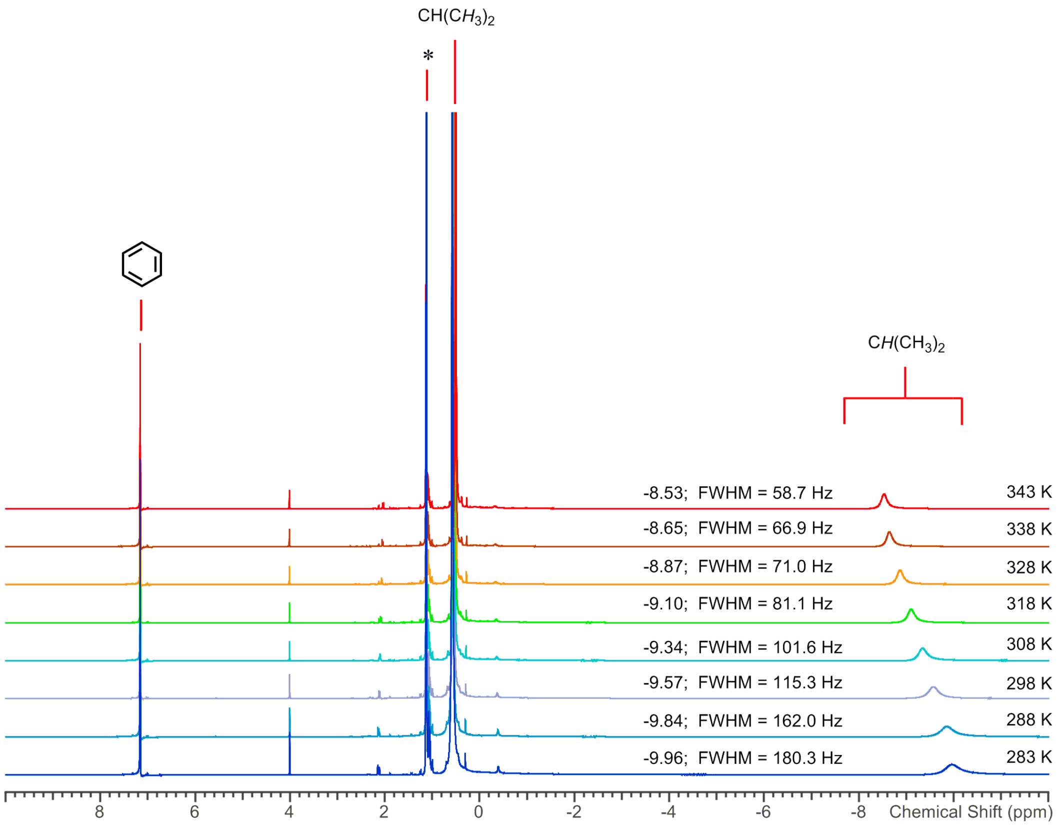

Fig. S5 Variable temperature (283 – 343 K) ^1H NMR spectra of **2-F** in C_6D_6 (* denotes $HN(Si^iPr_3)_2$)

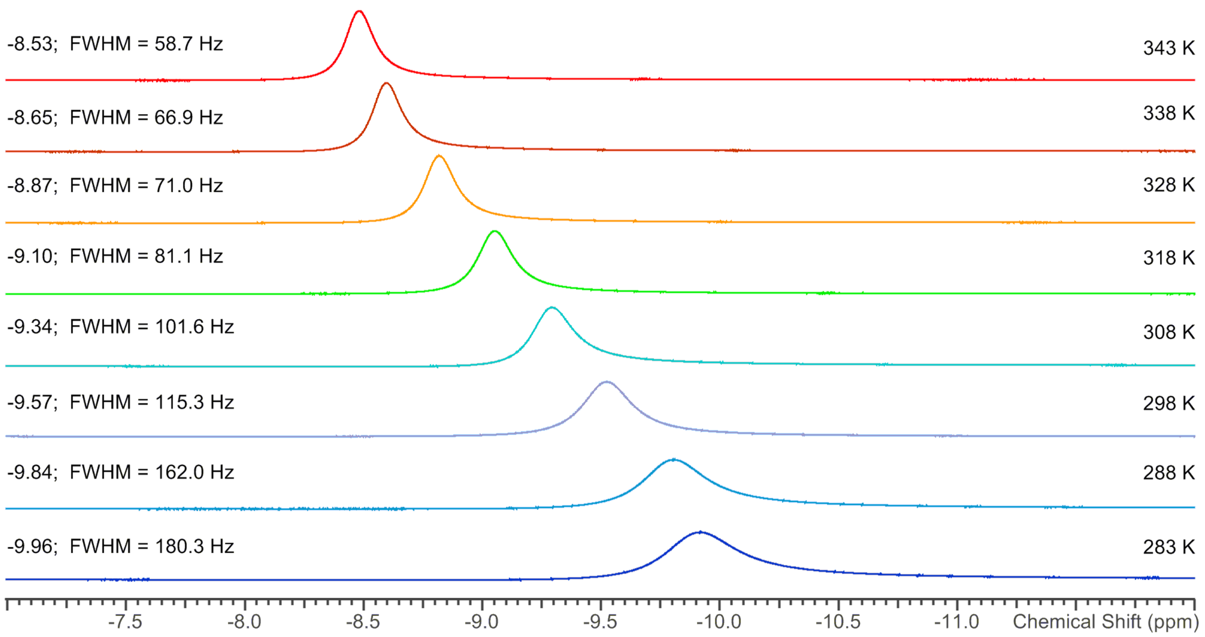

Fig. S6 Variable temperature (283 – 343 K) ^{1}H NMR spectra of **2-F** in C_6D_6 tracking the $CH(CH_3)_2$ resonance

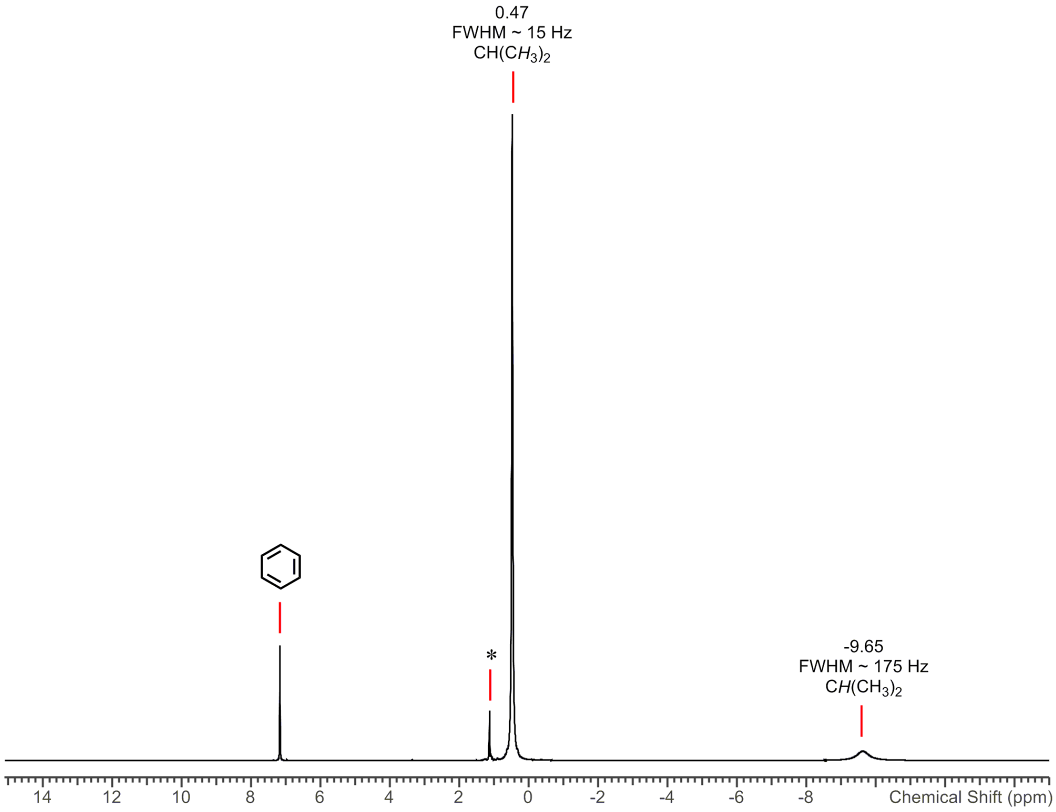

Fig. S7 ^{1}H NMR spectrum of **2-Cl** in C_6D_6 (* denotes $HN(Si^iPr_3)_2$.)

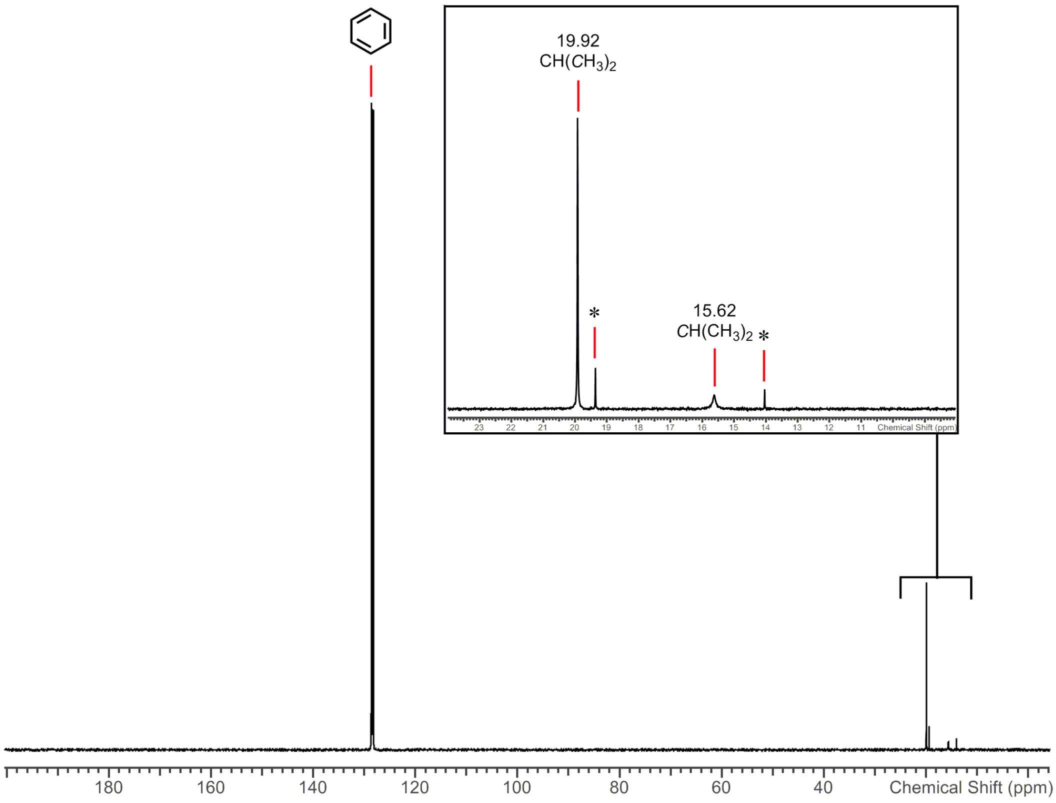

Fig. S8 ^{13}C{^{1}H} NMR spectrum of **2-Cl** in C_6D_6 (* denotes HN(Si^{i}Pr$_3$)$_2$.)

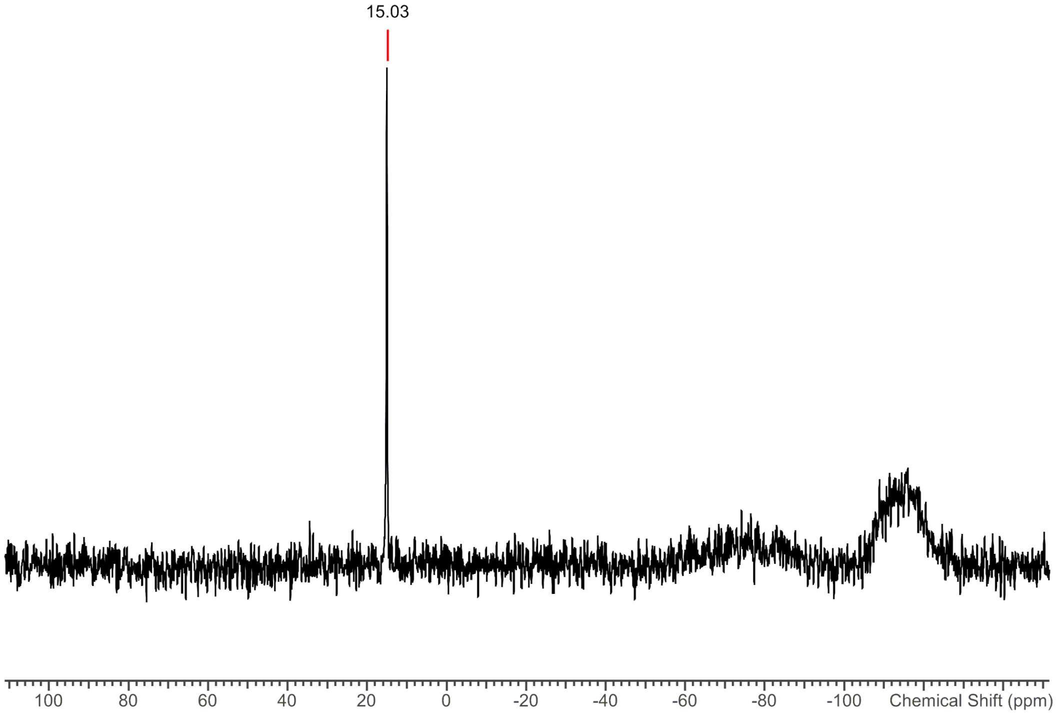

Fig. S9 ^{29}Si{^{1}H} NMR spectrum of **2-Cl** in C_6D_6. Broad features between -80 to -120 are glass.

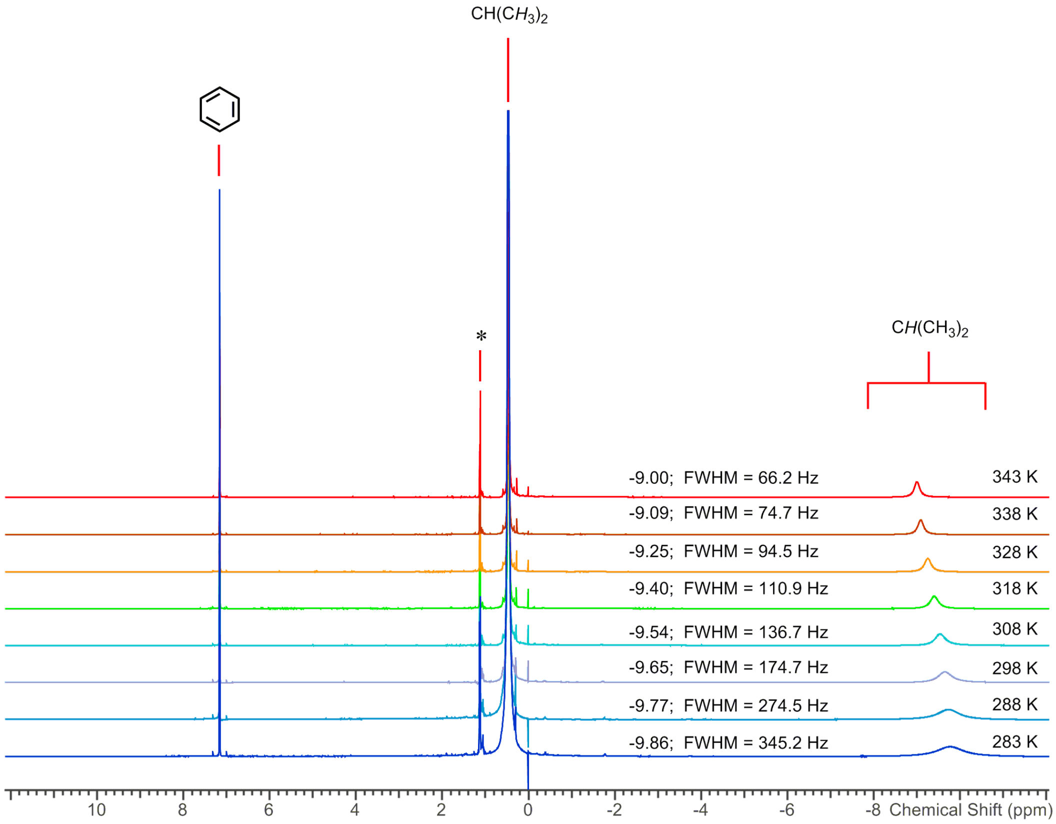

Fig. S10 Variable temperature (283 – 343 K) ^{1}H NMR spectra of **2-Cl** in C_6D_6 (* denotes $HN(Si^{i}Pr_3)_2$)

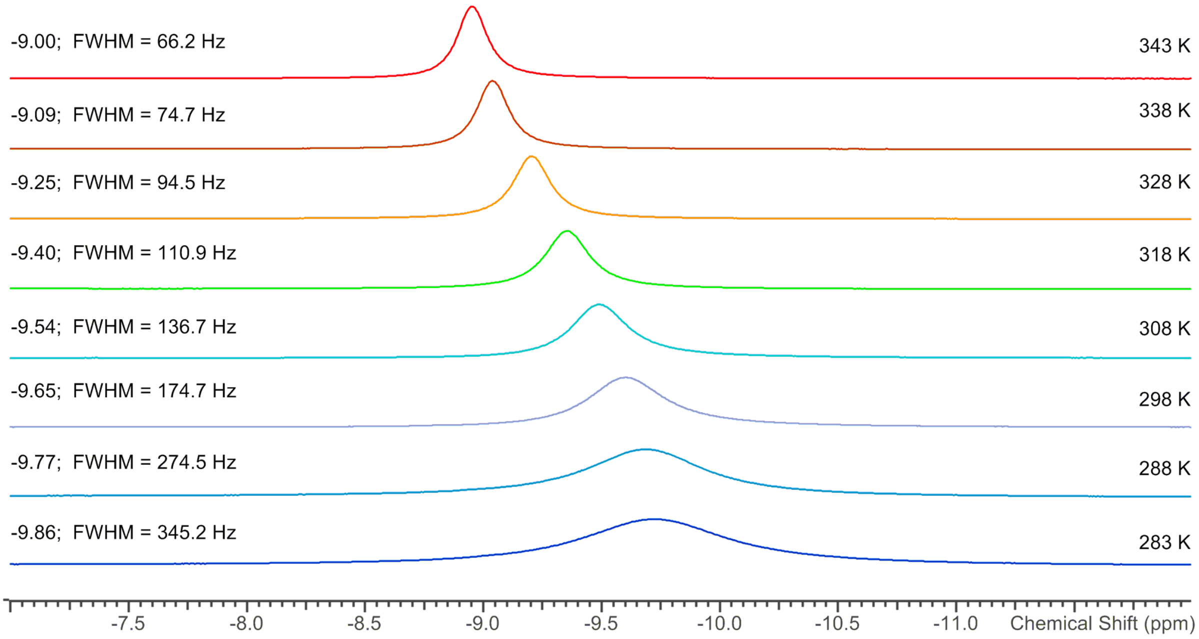

Fig. S11 Variable temperature (283 – 343 K) ^{1}H NMR spectra of **2-Cl** in C_6D_6 tracking the $C\mathit{H}(CH_3)_2$ resonance.

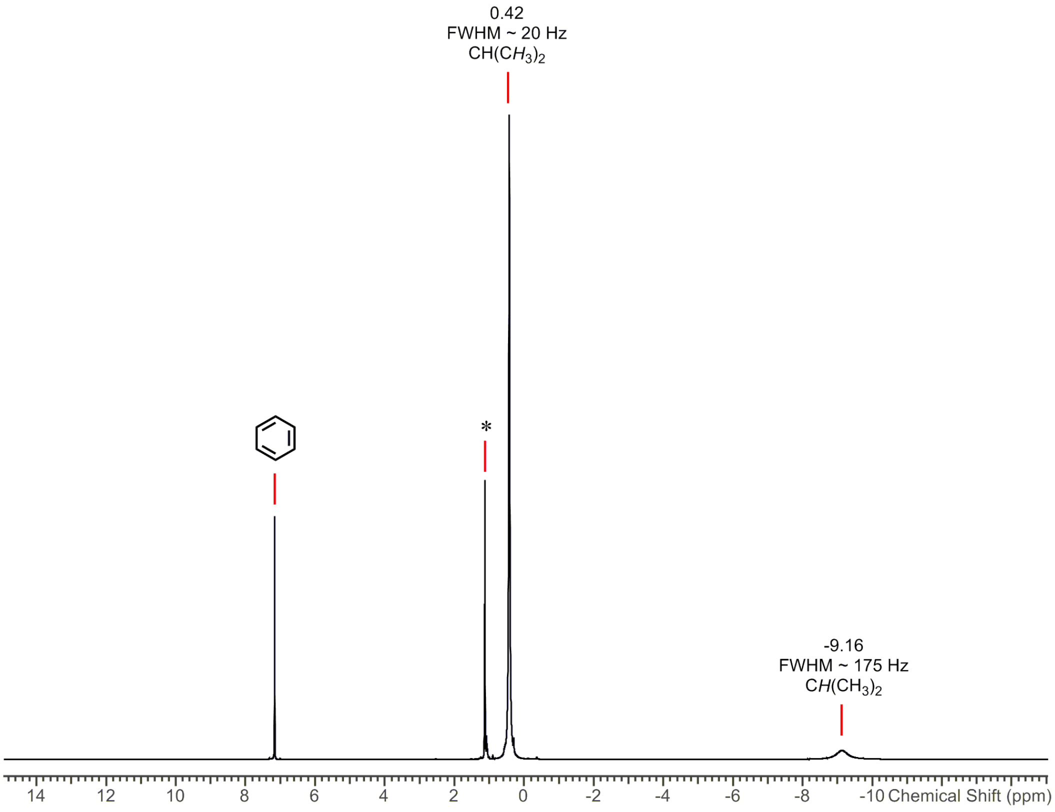

Fig. S12 ^{1}H NMR spectrum of **2-Br** in C_6D_6 (* denotes $HN(Si^iPr_3)_2$)

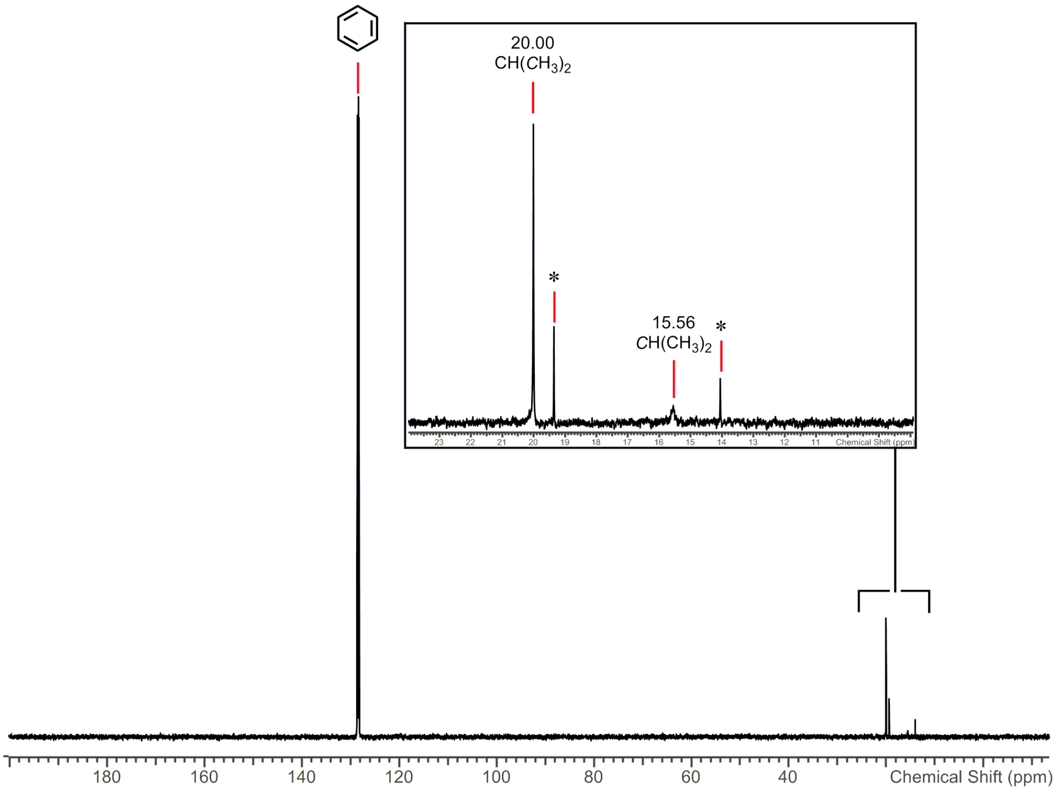

Fig. S13 ^{13}C{^{1}H} NMR spectrum of **2-Br** in C_6D_6 (* denotes $HN(Si^iPr_3)_2$)

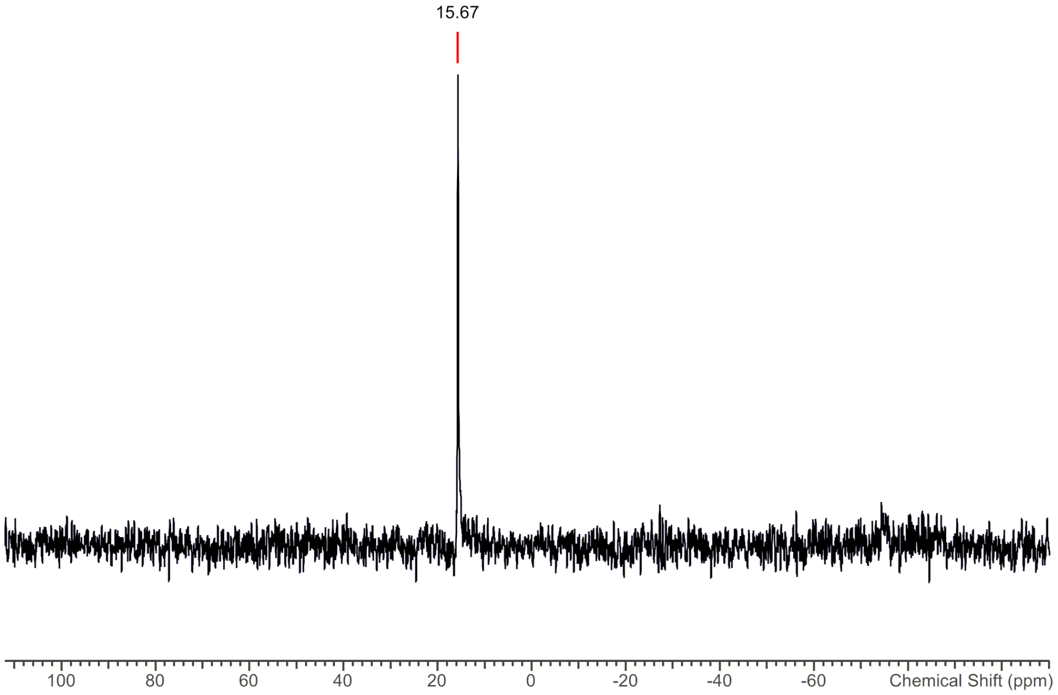

Fig. S14 $^{29}Si\{^{1}H\}$(DEPT) NMR spectrum of **2-Br** in C_6D_6

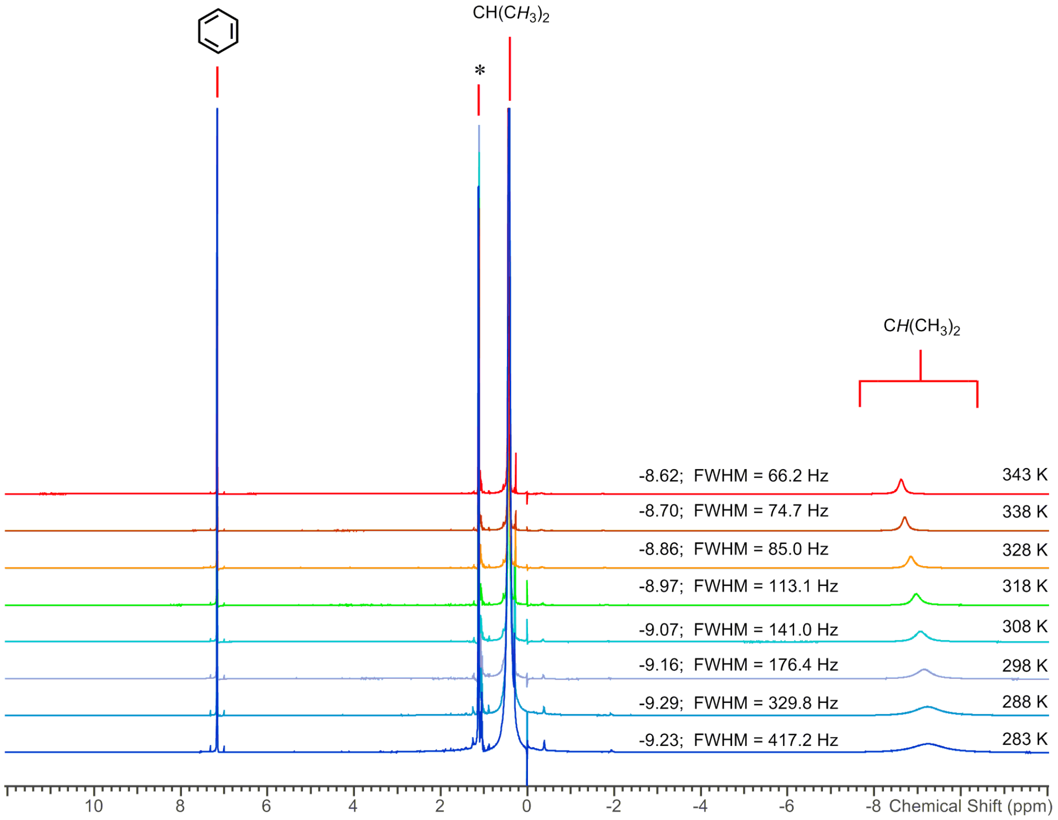

Fig. S15 Variable temperature (283 – 343 K) ^{1}H NMR spectra of **2-Br** in C_6D_6 (* denotes $HN(Si^{i}Pr_3)_2$)

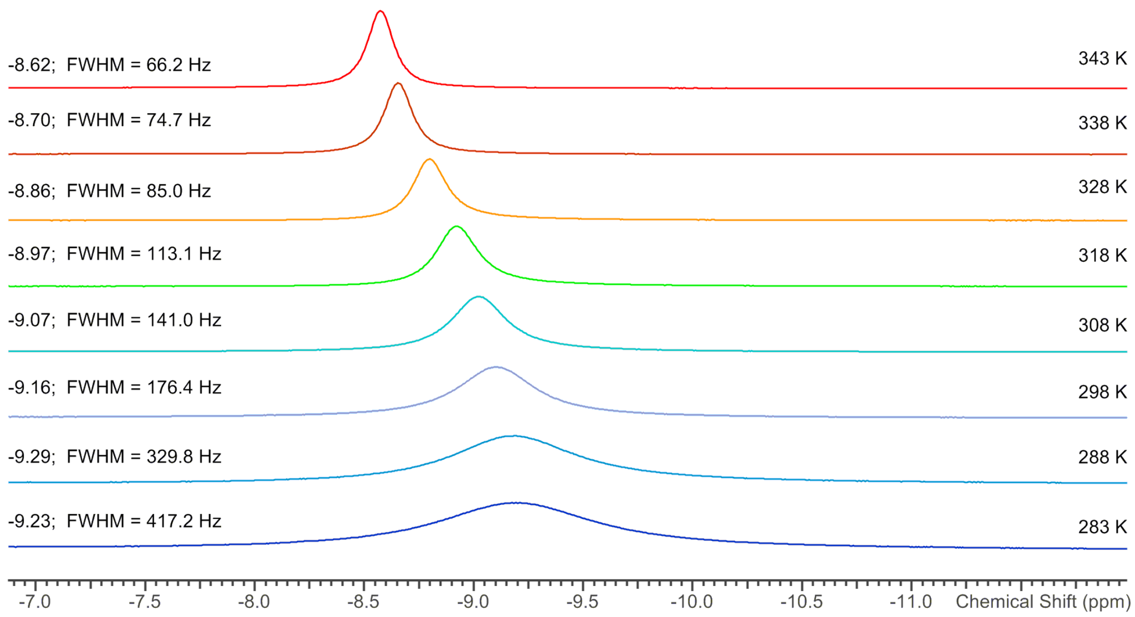

Fig. S16 Variable temperature (283 – 343 K) ^{1}H NMR spectra of **2-Br** in C_6D_6 tracking the C*H*(CH_3)$_2$ resonance.

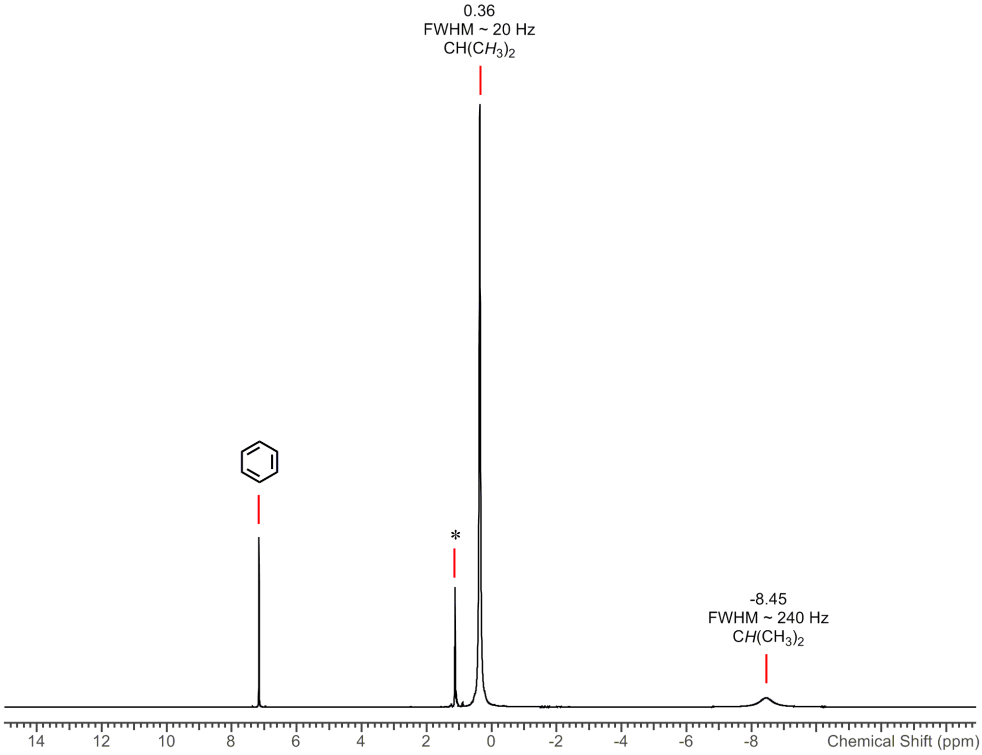

Fig. S17 ^{1}H NMR spectrum of **2-I** in C_6D_6 (* denotes HN(Si^{i}Pr$_3$)$_2$)

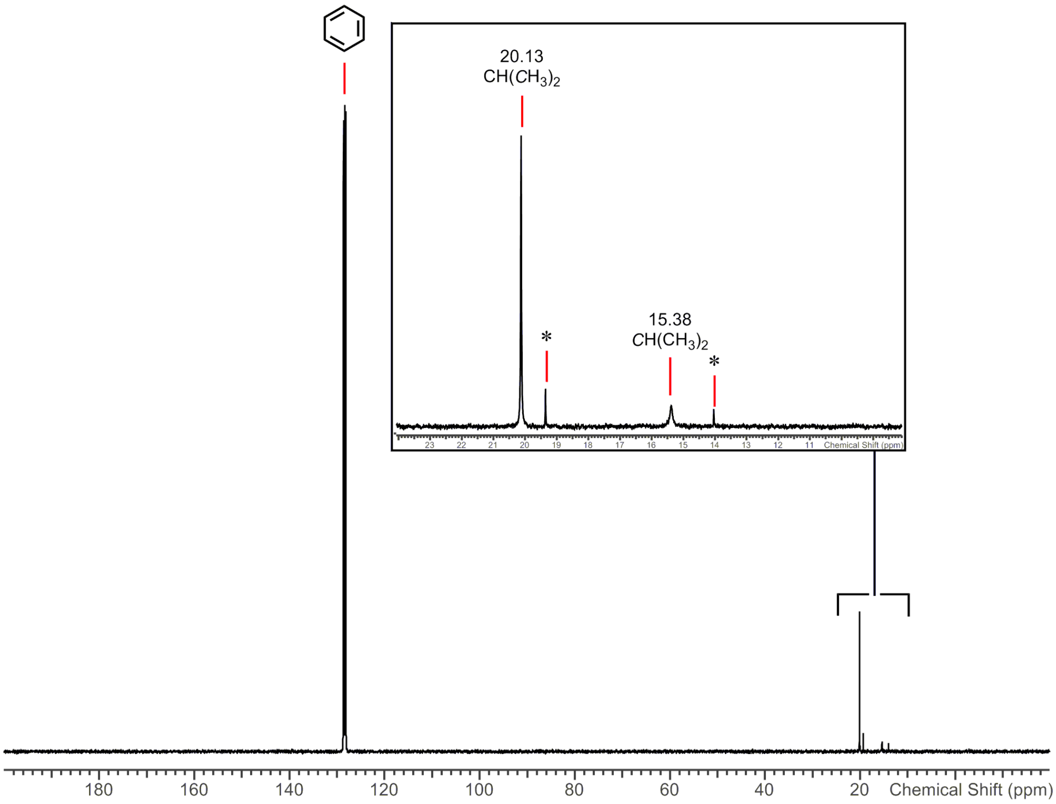

Fig. S18 $^{13}C\{^{1}H\}$ NMR spectrum of **2-I** in C_6D_6 (* denotes $HN(Si^{i}Pr_3)_2$)

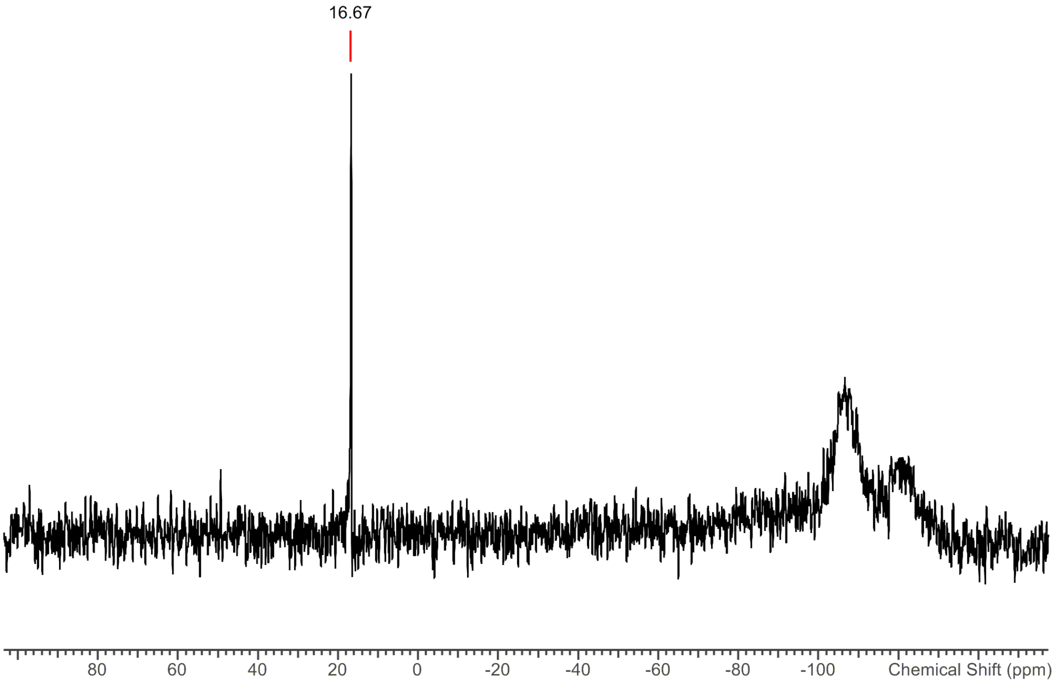

Fig. S19 $^{29}Si\{^{1}H\}$ NMR spectrum of **2-I** in C_6D_6. Broad features between -80 to -120 are glass.

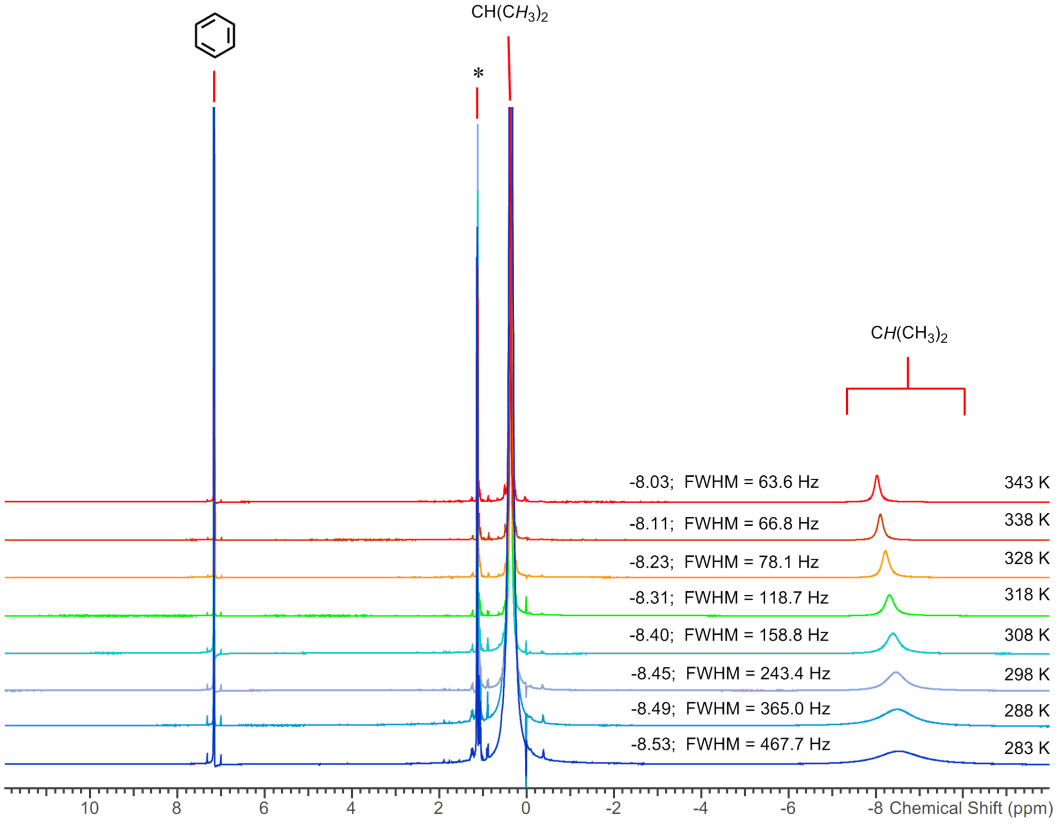

Fig. S20 Variable temperature (283 – 343 K) ¹H NMR spectra of **2-I** in C_6D_6 (* denotes $HN(Si^iPr_3)_2$)

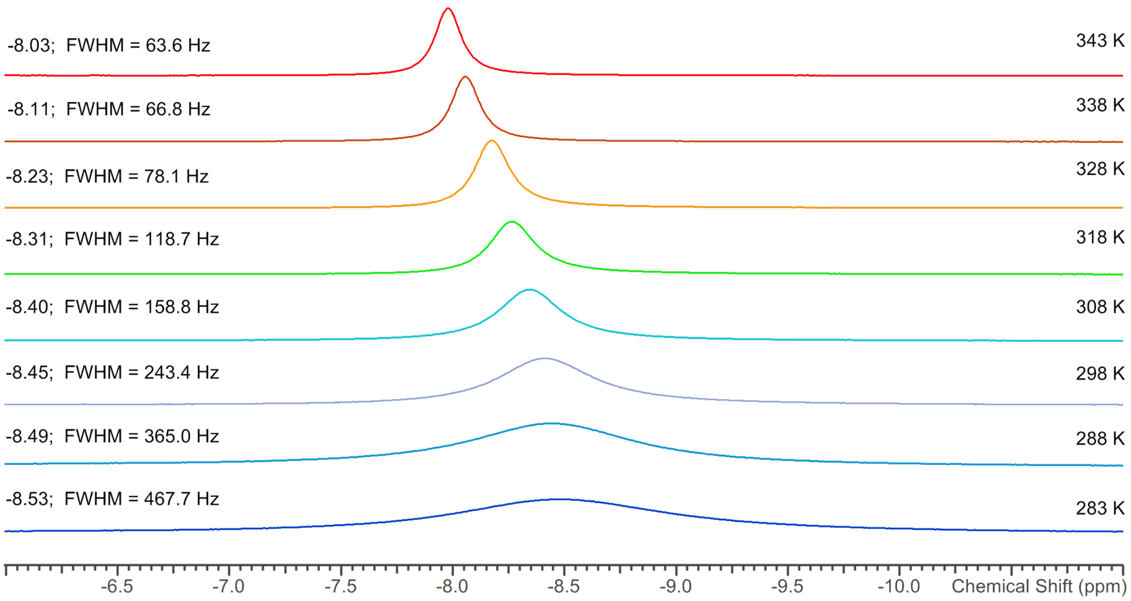

Fig. S21 Variable temperature (283 – 343 K) ¹H NMR spectra of **2-I** in C_6D_6 tracking the C*H*$(CH_3)_2$ resonance.

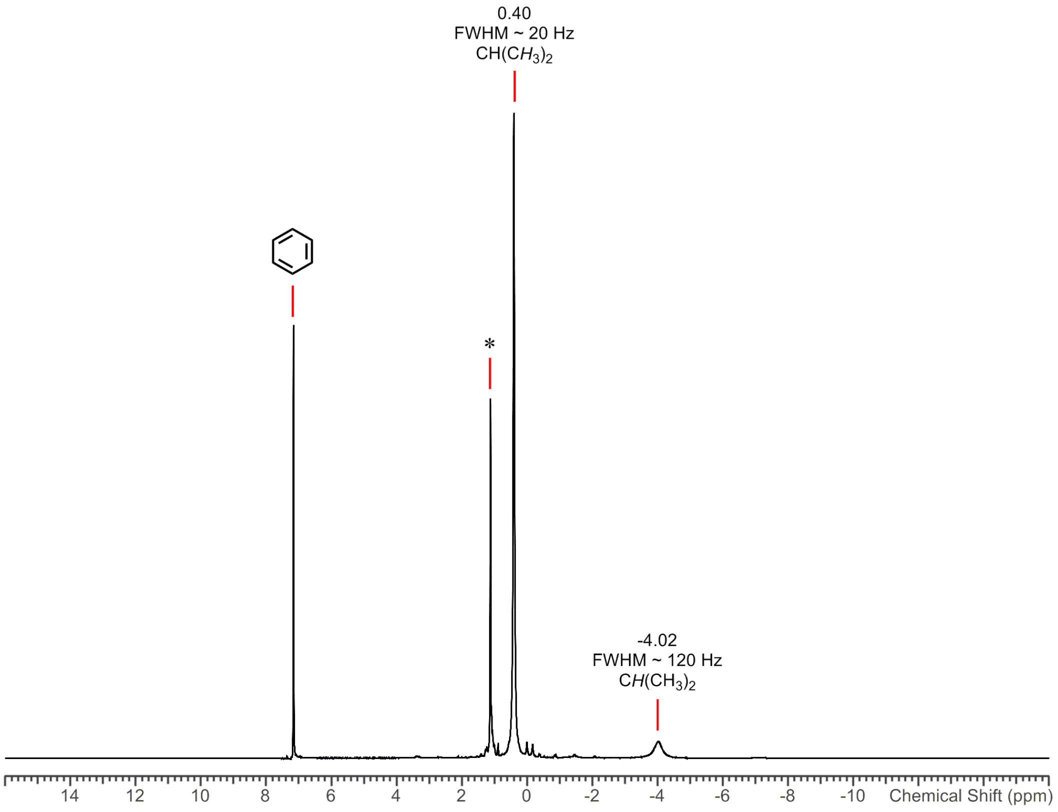

Fig. S22 ^{1}H NMR spectrum of **3** in C_6D_6 (* denotes $HN(Si^iPr_3)_2$)

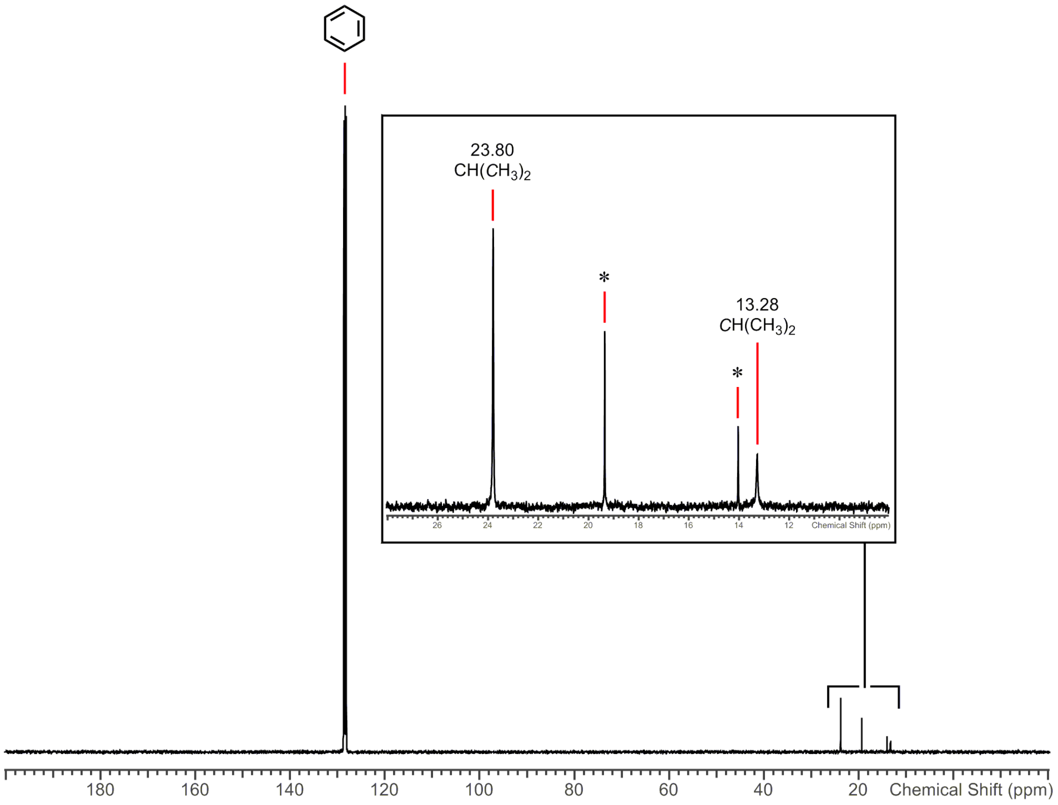

Fig. S23 ^{13}C{^{1}H} NMR spectrum of **3** in C_6D_6 (* denotes $HN(Si^iPr_3)_2$)

3. FTIR Spectroscopy

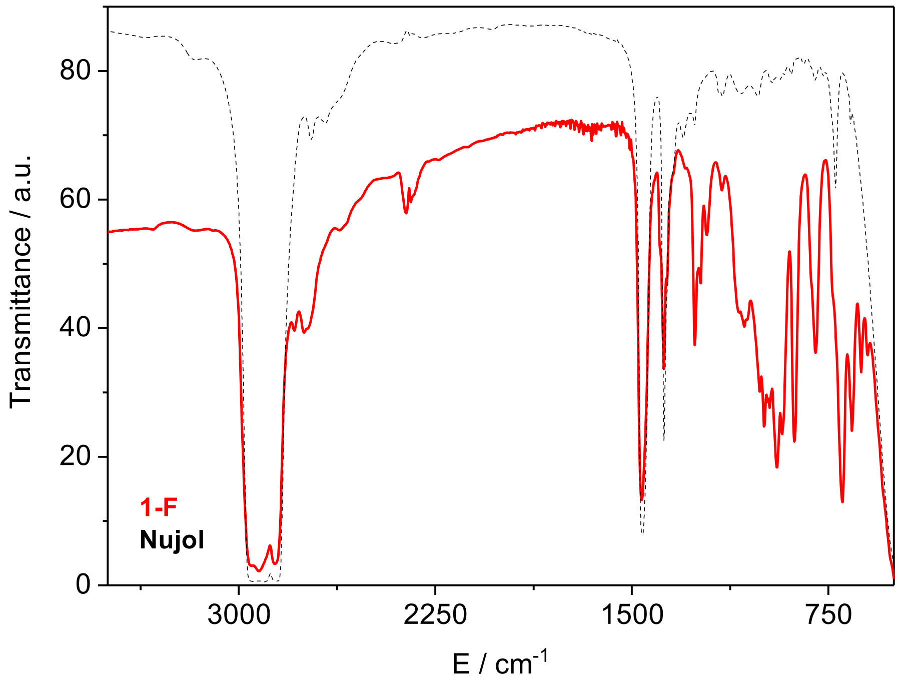

Fig. S24 FTIR spectrum of **2-F** as a Nujol mull on KBr discs recorded between 500–3500 cm^{-1}. Dotted trace is a spectrum of the Nujol intended to highlight overlaps.

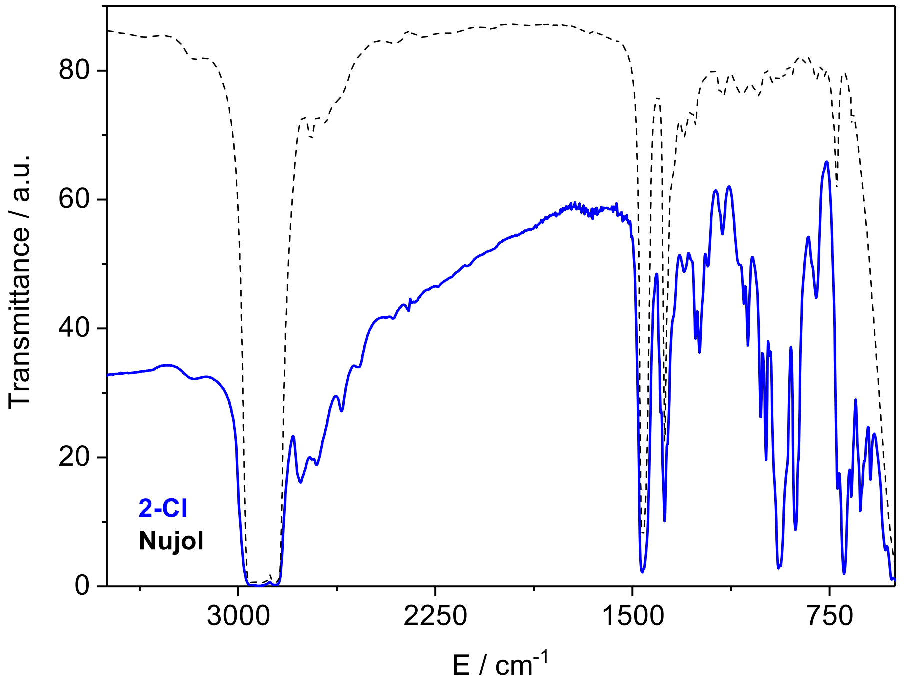

Fig. S25 FTIR spectrum of **2-Cl** as a Nujol mull on KBr discs recorded between 500–3500 cm^{-1}. Dotted trace is a spectrum of the Nujol intended to highlight overlaps.

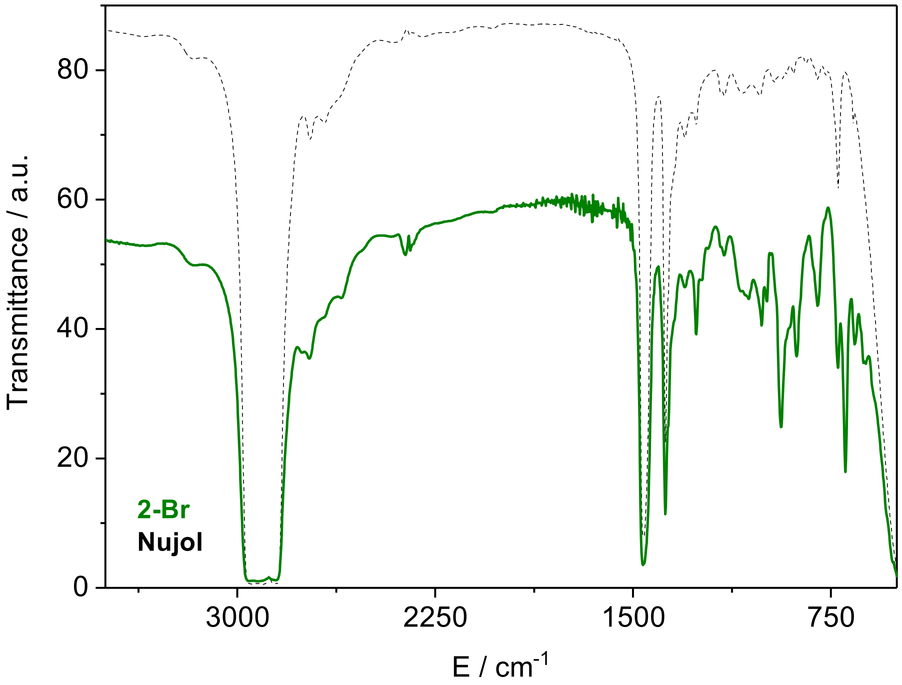

Fig. S26 FTIR spectrum of **2-Br** as a Nujol mull on KBr discs recorded between 500–3500 cm^{-1}. Dotted trace is a spectrum of the Nujol intended to highlight overlaps.

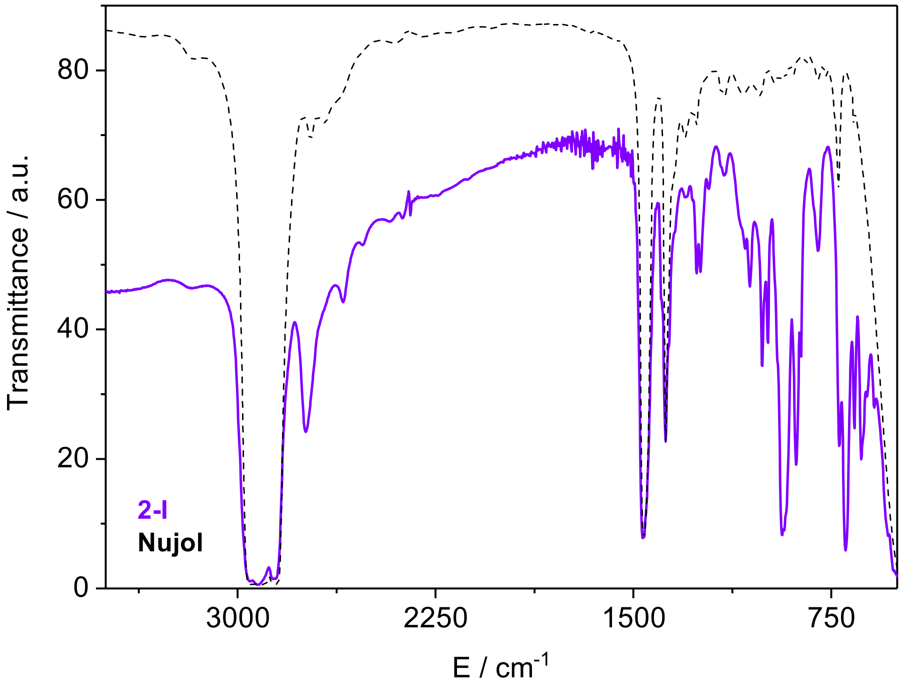

Fig. S27 FTIR spectrum of **2-I** as a Nujol mull on KBr discs recorded between 500–3500 cm^{-1}. Dotted trace is a spectrum of the Nujol intended to highlight overlaps.

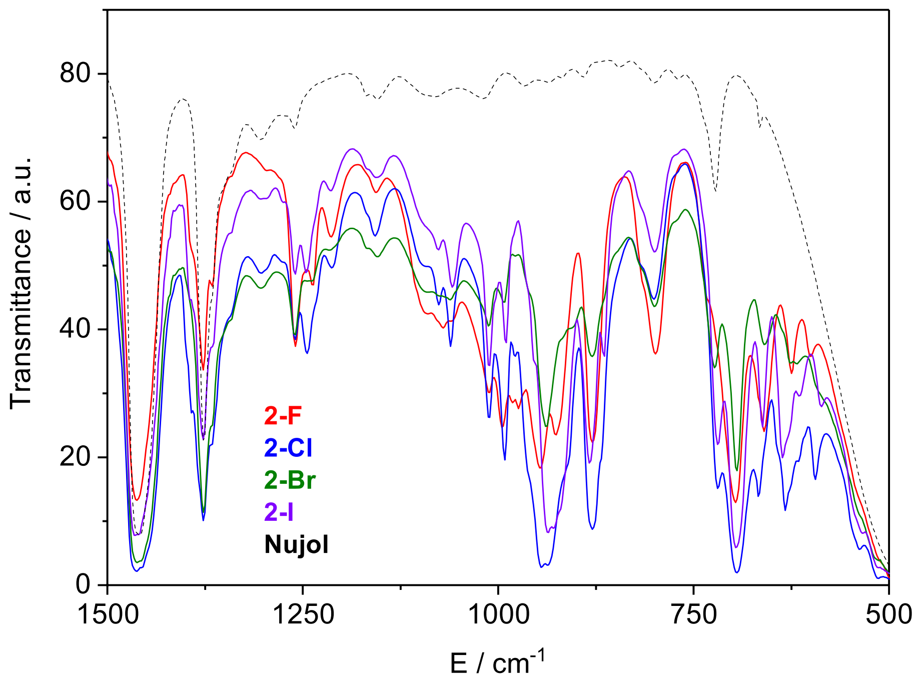

Fig. S28 FTIR spectrum of **2-X** as Nujol mulls on KBr discs recorded between 500–1500 cm^{-1}. Intended to show the similarity in the vibrational spectra of these isostructural complexes. Dotted trace is a spectrum of the Nujol intended to highlight overlaps.

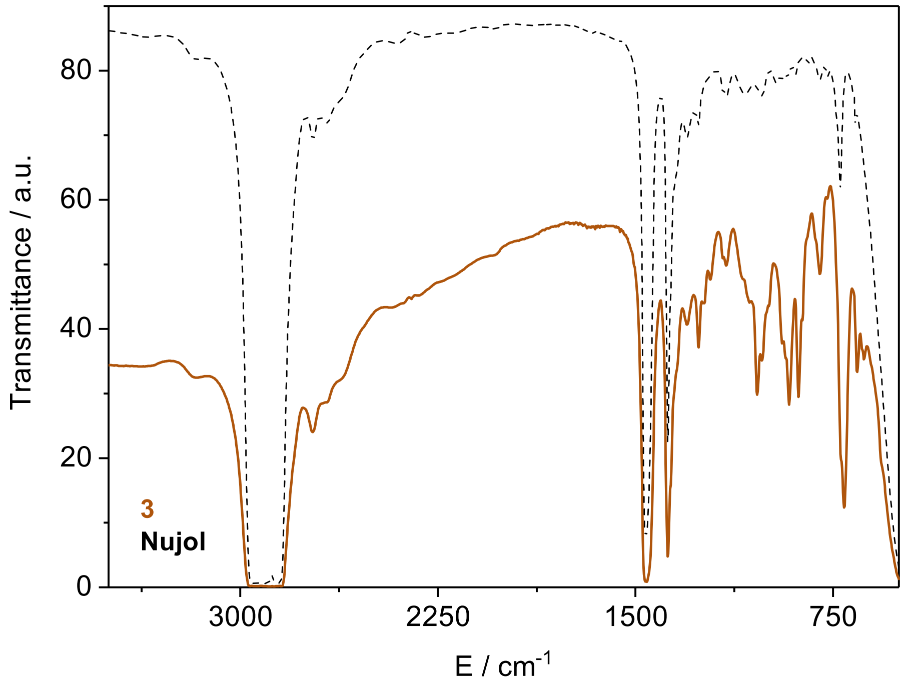

Fig. S29 FTIR spectrum of **3** as a Nujol mull on KBr discs recorded between 500–3500 cm^{-1}. Dotted trace is a spectrum of the Nujol intended to highlight overlaps.

4. Electronic Spectroscopy

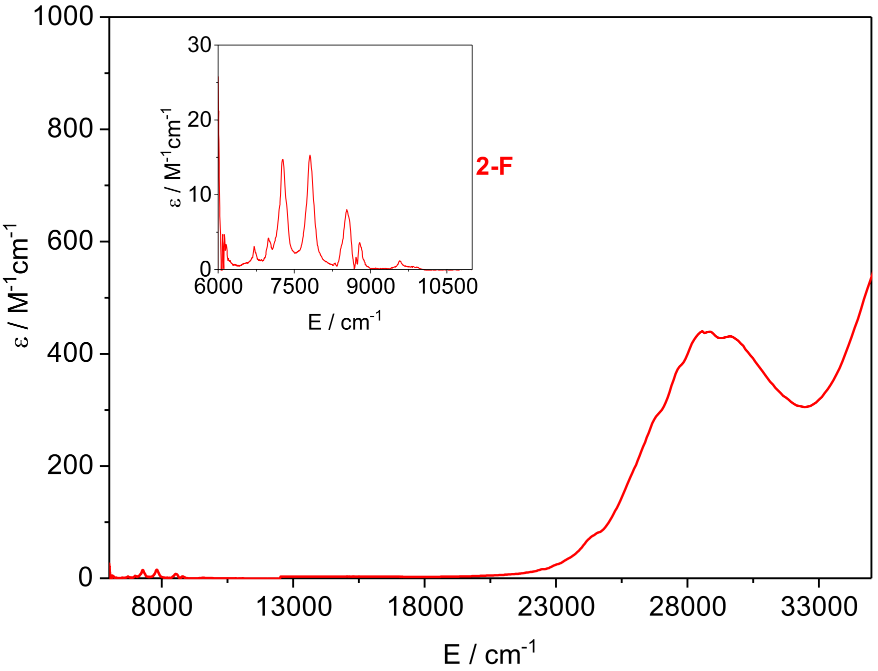

Fig. S30 Electronic spectrum of a toluene solution of **2-F** at ambient temperature. Inset shows expansion of the NIR region.

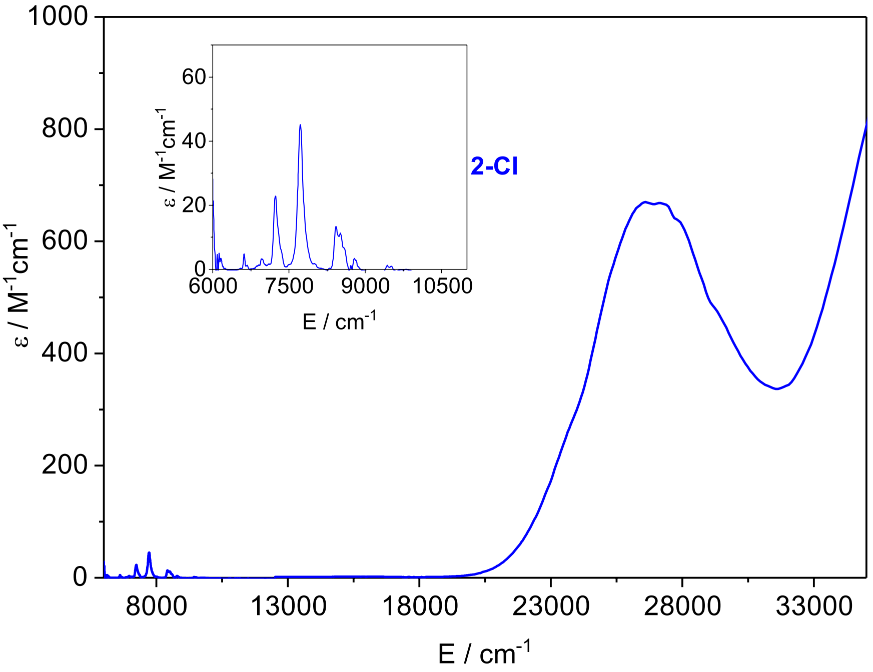

Fig. S31 Electronic spectrum of a toluene solution of **2-Cl** at ambient temperature. Inset shows expansion of the NIR region.

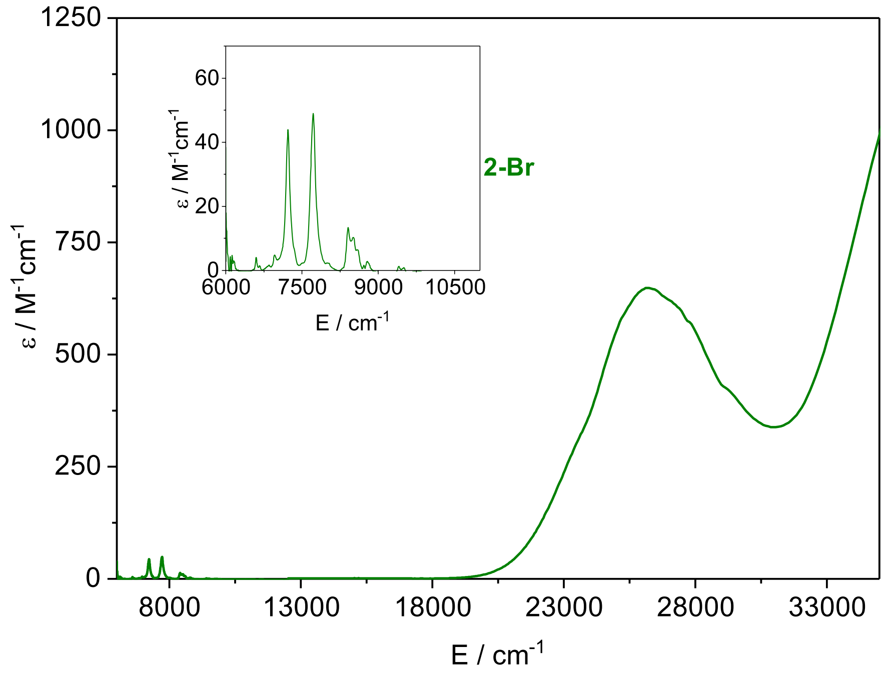

Fig. S32 Electronic spectrum of a toluene solution of **2-Br** at ambient temperature. Inset shows expansion of the NIR region.

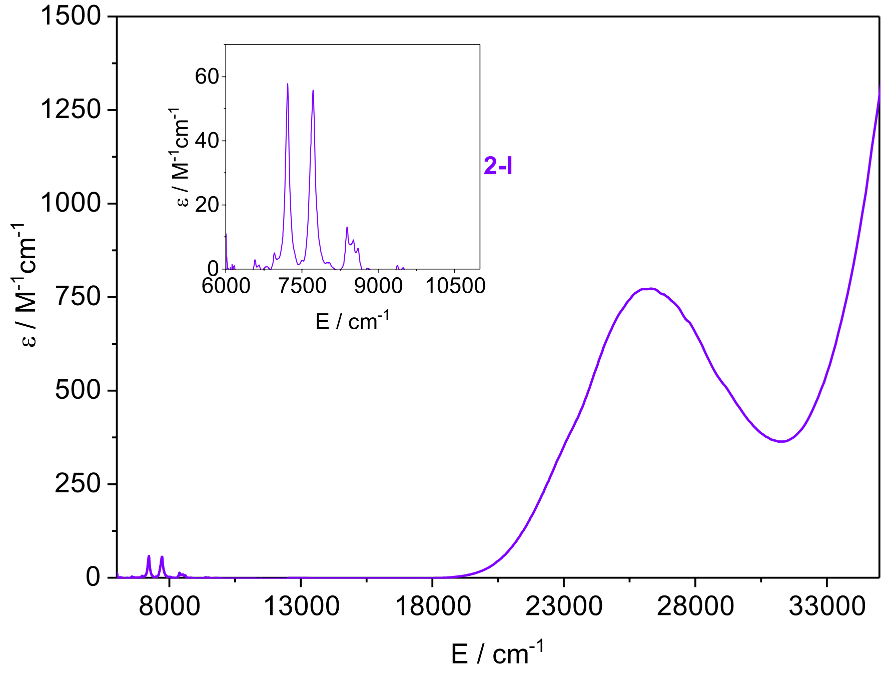

Fig. S33 Electronic spectrum of a toluene solution of **2-I** at ambient temperature. Inset shows expansion of the NIR region.

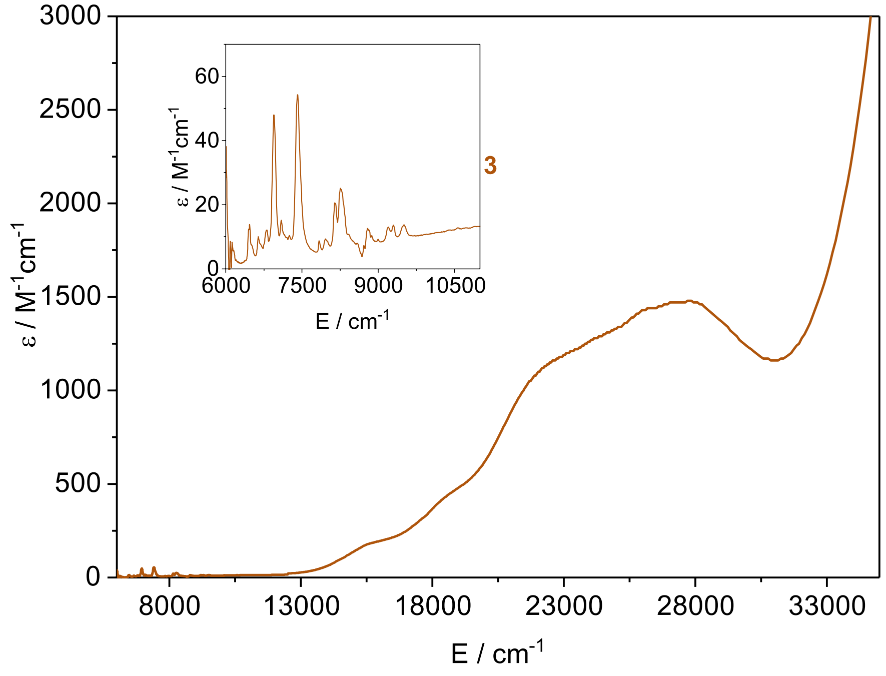

Fig. S34 Electronic spectrum of a toluene solution of **3** at ambient temperature. Inset shows expansion of the NIR region.

5. Crystallography

Diffraction quality single crystals of **2-F**, **2-I** and **3** were examined using an Oxford Diffraction Supernova diffractometer with a CCD area detector and a mirror-monochromated Mo Kα radiation (λ = 0.71073 Å). Crystals of **2-Cl** and **2-Br** were examined on an Oxford Diffraction Xcalibur diffractometer with a CCD area detector and a mirror-monochromated Mo Kα radiation (λ = 0.71073 Å). Intensities were recorded on 0.75° (**3**), 0.8° (**2-F**, **2-Cl** and **2-Br**), 0.9° (**2-I**) frames by ω rotation. Cell parameters were refined from the observed positions of all strong reflections in each data set. A Gaussian grid face-indexed absorption correction with beam profile modeling was applied in all instances.[1] The structures were by intrinsic phasing in SHELXT[2] and the datasets were refined by full-matrix least-squares on all unique F^2 values with anisotropic displacement parameters for all non-hydrogen atoms, and with constrained riding hydrogen geometries; U_{iso}(H) was set at 1.2 (1.5 for methyl groups) times U_{eq} of the parent atom. The largest features in final difference syntheses were close to heavy atoms and were of no chemical significance. CrysAlisPro[1] was used for control and integration, and SHELX[2,3] was employed through OLEX2[3] for structure solution and refinement. ORTEP-3[4] and POV-Ray[5] were employed for molecular graphics. Crystal data are compiled in Table S1.

Table S1 Crystallographic data for compounds **2-X** (X = F, Cl, Br, I) and **3**.

	**2-F**	**2-Cl**	**2-Br**	**2-I·0.5C$_6$H$_{14}$**	**3**
Formula	$C_{36}H_{84}FN_2Si_4Sm$	$C_{36}H_{84}ClN_2Si_4Sm$	$C_{36}H_{84}BrN_2Si_4Sm$	$C_{39}H_{91}IN_2Si_4Sm$	$C_{54}H_{126}I_5N_3Si_6Sm_3$
Formula weight	826.76	843.21	887.67	977.74	2071.66
Cryst size, mm	0.10 × 0.13 × 0.26	0.32 × 0.45 × 0.63	0.26 × 0.36 × 0.49	0.15 × 0.23 × 0.38	0.04 × 0.05 × 0.10
Crystal system	Triclinic	Monoclinic	Orthorhombic	Orthorhombic	Orthorhombic
Space group	*P*–1	$P2_1/c$	*Pbca*	*Pccn*	$Pna2_1$
a / Å	8.7196(2)	15.8726(4)	20.6319(9)	20.0340(4)	21.5645(13)
b / Å	10.9283(3)	13.1782(3)	20.1603(11)	20.2409(4)	40.200(2)
c / Å	25.1607(8)	22.6012(6)	21.9662(10)	24.0385(5)	26.9282(16)
α / °	91.865(2)	90	90	90	90
β / °	98.509(2)	109.299(3)	90	90	90
γ / °	112.973(3)	90	90	90	90
V / Å^3	2172.4(1)	4461.9(2)	9136.7(8)	9747.8(3)	23 344(2)
ρ_{calcd} / g cm^3	1.264	1.255	1.291	1.332	1.768
Z	2	4	8	8	12
μ / mm^{-1}	1.491	1.508	2.289	1.963	4.342
$F(000)$	882	1796	3736	4080	12 072
no. of reflns (unique)	15 060 (7965)	22 398 (8147)	32 776 (8310)	39 822 (8929)	175 110 (42 706)
S^a	1.05	1.06	1.04	1.05	0.97
$R_1(wR_2)$ ($F^2 > 2\sigma(F^2)$)	0.0400 (0.0837)	0.0286 (0.0658)	0.0459 (0.1063)	0.0342 (0.0731)	0.1258 (0.2969)
R_{int}	0.036	0.032	0.068	0.048	0.445
min./max. diff map / Å^{-3}	–0.49, 1.07	–0.49, 0.84	–1.12, 0.70	–0.56, 1.17	–1.30, 2.25

[a] Conventional $R = \Sigma||F_o| - |F_c||/\Sigma|F_o|$; $R_w = [\Sigma w(F_o^2 - F_c^2)^2/\Sigma w(F_o^2)^2]^{1/2}$; $S = [\Sigma w(F_o^2 - F_c^2)^2/\text{no. data} - \text{no. params})]^{1/2}$ for all data.

6. X-ray Absorption Spectroscopy

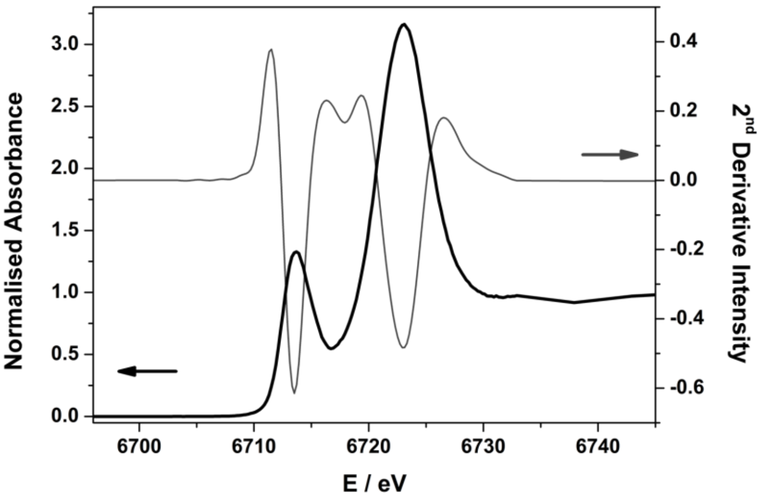

Fig. S35 Overlay of the normalised Sm L_3-edge spectrum of **1** with its FFT-smoothed second derivative spectrum.

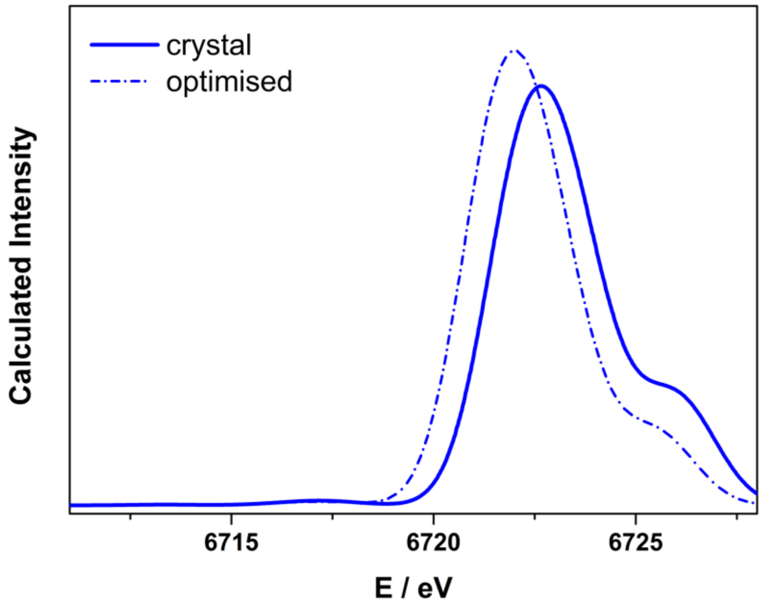

Fig. S36 Overlay of the TD-DFT calculated Sm L_3-edge spectrum of **2-Cl** comparing the structure determined by crystallography (solid line) with the geometry optimised structure (dashed line). Spectra have been shifted +78.6 eV to match the experimental data.

Table S2 Comparison of the Experimental and Optimised Metrics for **2-Cl**

	**Experimental**	**Calculated**
Sm–Cl	2.5813(7)	2.662
Sm–N_{avg}	2.306(3)	2.409
N–Sm–N	128.24(7)	129.9
N–Sm–Cl_{avg}	114.23(8)	114.4
Sm···N_2Cl_{plane}	0.250(2)	0.171

Table S3 Geometry Optimised Coordinates of **2-Cl**

Sm	0.00000	0.00000	0.00000
Cl	-0.00000	-0.00000	2.66167
Si	-1.02663	2.57602	-2.14227
Si	1.09379	3.46349	0.02686
Si	-0.94064	-3.23231	-0.72870
Si	1.85630	-2.23995	-1.95361
N	0.13728	2.26989	-0.86247
N	0.32583	-2.08228	-1.11711
C	-2.16446	1.03474	-2.25375
C	-0.14916	2.76343	-3.82445
C	-2.15306	4.08417	-1.85903
C	2.51208	2.55926	0.91515
C	1.84735	4.76412	-1.14984
C	0.03166	4.35079	1.33576
C	-2.31268	-2.25429	0.18719
C	-1.70958	-4.02117	-2.28308
C	-0.34304	-4.61085	0.43854
C	2.87344	-0.67043	-1.53772
C	1.61507	-2.30279	-3.84314
C	2.87785	-3.75271	-1.41639
H	-2.87852	1.16370	-3.08913
H	-2.77943	0.89968	-1.34162
H	-1.61862	0.09284	-2.46542
H	-0.89193	2.86973	-4.63810
H	0.47959	1.88169	-4.04226
H	0.50070	3.65640	-3.83650
H	-2.89318	4.16886	-2.67754
H	-1.57411	5.02491	-1.83343
H	-2.70565	3.99820	-0.90601
H	3.18492	2.05323	0.19740
H	2.13227	1.81283	1.64073
H	3.12349	3.27871	1.49159
H	1.06830	5.35618	-1.66282
H	2.48047	4.28632	-1.91967
H	2.48016	5.46702	-0.57607
H	-0.40318	3.61782	2.03898
H	-0.79273	4.92470	0.87612
H	0.65701	5.05431	1.91680
H	-2.77132	-1.46937	-0.44547
H	-1.97119	-1.80607	1.14369
H	-3.13129	-2.94619	0.46082
H	-2.09798	-3.25387	-2.97725
H	-2.54755	-4.68636	-2.00147
H	-0.96456	-4.62923	-2.82788
H	-1.18847	-5.26113	0.73240
H	0.09419	-4.18217	1.35792
H	0.42286	-5.24471	-0.04236
H	3.83704	-0.70047	-2.08054
H	3.12482	-0.60452	-0.46089
H	2.37893	0.27280	-1.84653
H	1.05922	-1.41853	-4.20457
H	1.04918	-3.20404	-4.14078
H	2.59282	-2.33025	-4.36048
H	2.37968	-4.69672	-1.70085
H	3.03100	-3.76428	-0.32266
H	3.87147	-3.73817	-1.90262

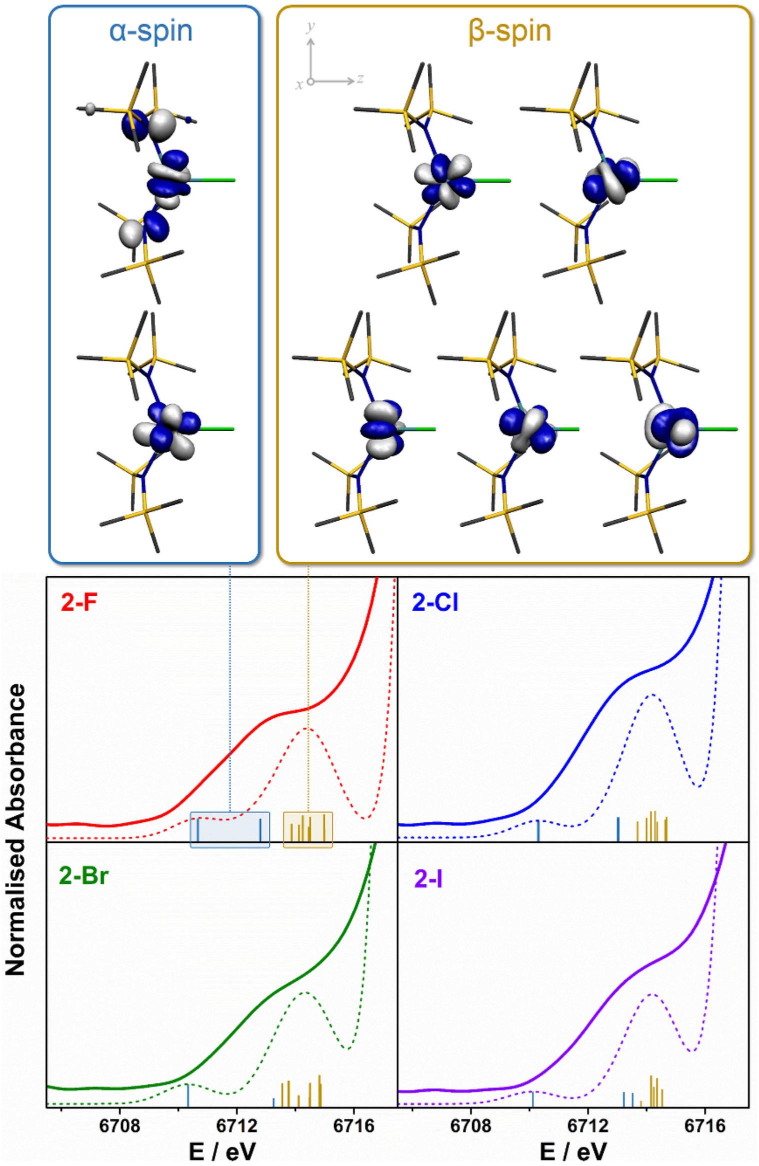

Fig. S37 Assignment of the pre-edge region of the Sm L_3-edge spectra of **2-X** (X = F, Cl, Br, I). Experimental data are shown by the solid line; calculated pre-edge spectra are represented by the dashed trace and have been shifted to align with the experimental data. Blue vertical bars depict the α-spin electronic transitions to acceptor orbitals (unrestricted natural orbitals) shown in the box top left; yellow vertical bars depict the β-spin electronic transitions to acceptor orbitals (unrestricted natural orbitals) shown top right, which are the five SOMOs of the Sm(III) f^5 centre.

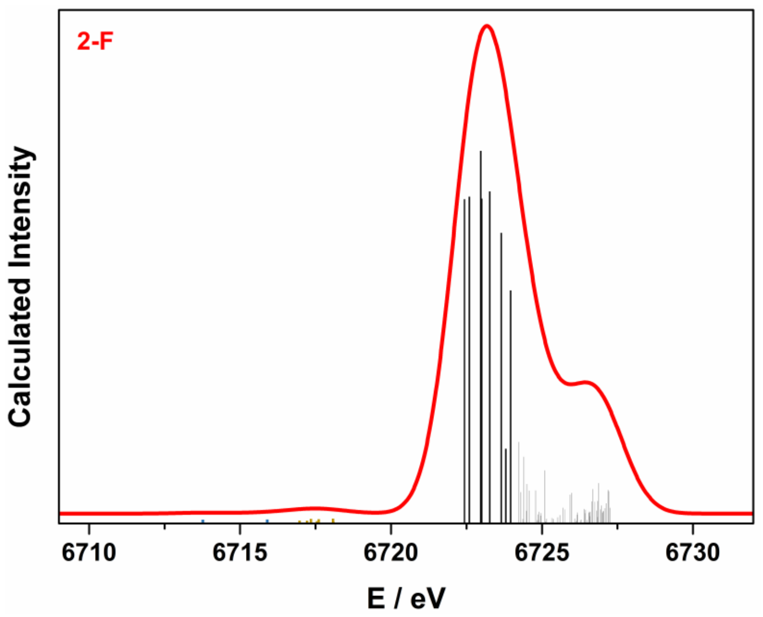

Fig. S38 Simulated Sm L_3-edge spectrum of **2-F** (red) and stick plot showing the individual transitions that comprise the spectrum. Colour palette: azure, 4f (α-spin); gold, 4f (β-spin) (see Fig. S37 for enlargement); black, 5d and 6s. Grey transitions lie beyond the edge jump. Details of the acceptor MOs comprising each transition are given in Table S4.

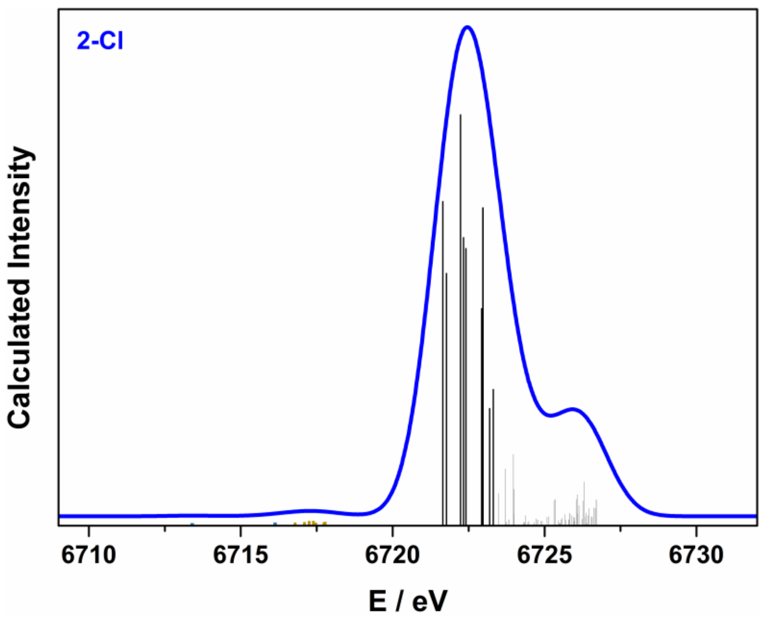

Fig. S39 Simulated Sm L_3-edge spectrum of **2-Cl** (blue) and stick plot showing the individual transitions that comprise the spectrum. Colour palette: azure, 4f (α-spin); gold, 4f (β-spin) (see Fig. S37 for enlargement); black, 5d and 6s. Grey transitions lie beyond the edge jump. Details of the acceptor MOs comprising each transition are given in Table S6.

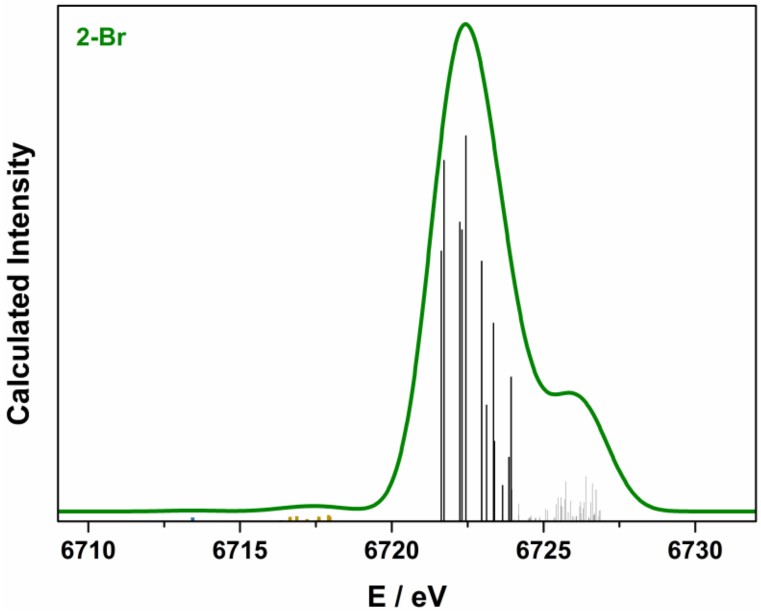

Fig. S40 Simulated Sm L_3-edge spectrum of **2-Br** (green) and stick plot showing the individual transitions that comprise the spectrum. Colour palette: azure, 4f (α-spin); gold, 4f (β-spin) (see Fig. S37 for enlargement); black, 5d and 6s. Grey transitions lie beyond the edge jump. Details of the acceptor MOs comprising each transition are given in Table S8.

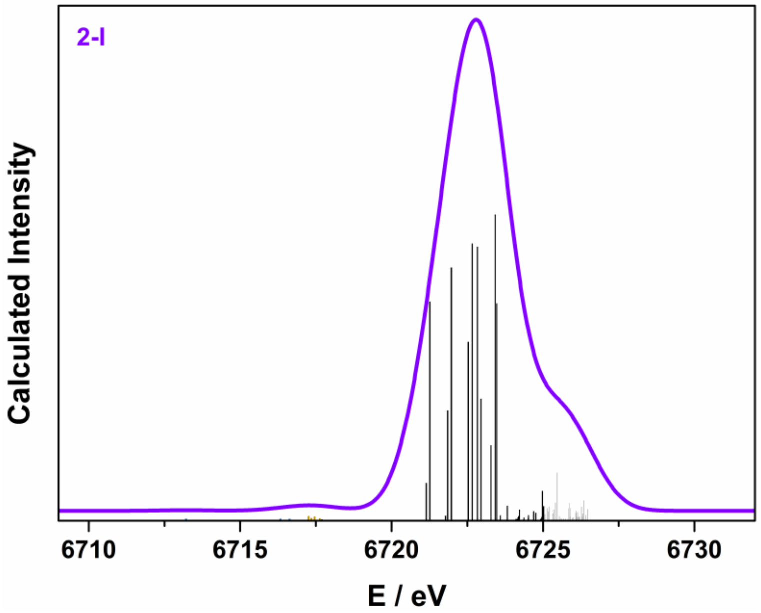

Fig. S41 Simulated Sm L_3-edge spectrum of **2-I** (violet) and stick plot showing the individual transitions that comprise the spectrum. Colour palette: azure, 4f (α-spin); gold, 4f (β-spin) (see Fig. S37 for enlargement); black, 5d and 6s. Grey transitions lie beyond the edge jump. Details of the acceptor MOs comprising each transition are given in Table S10.

Table S4 Acceptor Orbital Composition of TD-DFT Calculated Transitions in **2-F**

**Transition**[a]	**Acceptor MO**[b]
1	128a
2	127a
3	134b / 133b / 135b / 136b
4	136b / 138b
5	139b / 137b / 142b
6	144b / 140b / 143b / 142b
7	141b / 143b
8	149b / 148b / 150b
9	150b / 151b / 153b / 145b
10	134a / 131a / 132a
11	133a / 135a
12	140a
13	130b / 140b / 122b / 125b
14	128b / 132b / 131b
15	122b / 135b
16	129a
17	122b / 125b

[a] Colour palette: azure, 4f (α-spin); gold, 4f (β-spin); black, 5d and 6s (see Fig. S38). [b] Greater than 10% contribution to transition. Orbital composition is given in Table S5.

Table S5 Sm Contribution to Acceptor MOs in **2-F**

MO	4f	5d	6p	6s
127a	26.7	0.7	0.3	0.0
128a	95.5	0.6	0.1	0.4
129a	2.1	7.3	19.1	26.1
131a	2.1	22.5	41.3	1.2
132a	0.7	24.4	37.4	2.6
133a	1.3	62.2	1.4	1.1
134a	0.7	35.1	10.4	4.0
135a	1.6	54.8	6.0	2.1
140a	0.9	42.0	4.5	1.4
122b	2.2	5.8	20.4	26.1
125b	2.2	24.9	29.1	6.4
128b	6.3	38.6	4.9	0.9
130b	1.9	43.7	4.1	1.3
131b	9.7	27.3	15.0	1.5
132b	9.1	38.9	9.5	2.4
133b	29.2	21.9	5.8	2.1
134b	25.0	14.4	2.1	1.3
135b	17.6	13.0	3.6	1.7
136b	43.3	12.7	4.3	0.1
137b	18.1	14.6	4.1	1.0
138b	30.0	9.6	3.1	0.2
139b	28.6	9.7	4.6	0.6
140b	43.7	4.7	2.4	1.1
141b	41.9	5.6	2.4	0.0
142b	29.5	12.9	6.0	0.8
143b	34.1	7.6	1.2	0.4
144b	42.3	4.8	0.7	1.1
145b	24.5	10.6	2.2	0.8
148b	9.9	11.6	5.5	0.4
149b	43.8	10.6	4.7	0.6
150b	44.6	4.3	5.2	0.8
151b	17.7	15.6	6.5	0.2
153b	24.7	9.2	1.3	0.1

Table S6 Acceptor Orbital Composition of TD-DFT Calculated Transitions in **2-Cl**

**Transition**[a]	**Acceptor MO**[b]
1	132a
2	131a
3	141b / 138b / 143b
4	144b / 143b / 147b
5	148b / 150b / 142b
6	151b / 146b / 152b
7	148b / 151b / 153b
8	155b
9	161b / 156b
10	134a / 137a
11	137a / 138a
12	144a / 138a
13	133b / 129b / 126b
14	131b / 130b
15	138b / 133b / 126b
16	133a / 143a / 141a
17	126b / 136b
18	146a / 148a

[a] Colour palette: azure, 4f (α-spin); gold, 4f (β-spin); black, 5d and 6s (see Fig. S39). [b] Greater than 10% contribution to transition. Orbital composition is given in Table S7.

Table S7 Sm Contribution to Acceptor MOs in **2-Cl**

MO	4f	5d	6p	6s
131a	17.5	0.8	0.3	0.0
132a	97.0	0.3	0.0	0.2
133a	2.0	7.1	20.4	22.4
134a	2.5	38.5	13.7	1.8
137a	1.6	53.9	8.9	0.4
138a	0.5	41.0	8.6	4.1
141a	0.5	31.6	9.6	0.1
143a	2.9	36.8	11.4	5.7
144a	0.9	49.2	2.9	0.3
146a	1.9	23.8	6.8	0.4
148a	1.0	25.3	5.3	0.4
126b	2.2	5.0	22.9	22.6
129b	2.4	27.6	21.1	5.2
130b	2.0	62.2	1.6	1.8
131b	3.0	40.1	4.1	0.9
133b	1.6	42.6	6.1	1.6
136b	6.5	24.8	14.3	4.3
138b	7.6	29.6	4.1	3.5
141b	37.6	10.5	4.3	0.1
142b	10.5	9.6	5.0	0.4
143b	24.9	10.3	2.0	0.2
144b	35.0	6.2	5.6	1.0
146b	22.3	7.3	2.7	1.7
147b	41.7	10.1	0.8	1.4
148b	46.9	7.9	4.1	0.3
151b	43.0	12.7	2.2	0.4
152b	25.6	5.1	4.9	0.2
153b	46.7	8.1	4.9	0.7
155b	48.9	5.9	1.5	0.5
156b	18.6	8.5	2.0	0.3
161b	28.5	7.6	0.1	0.6

Table S8 Acceptor Orbital Composition of TD-DFT Calculated Transitions in **2-Br**

**Transition**[a]	**Acceptor MO**[b]
1	141a
2	140a
3	149b / 150b
4	150b / 152b / 153b
5	154b / 151b / 155b
6	159b / 158b / 162b
7	156b / 158b / 160b
8	165b / 162b
9	167b / 162b / 168b
10	142a / 143a
11	144a
12	138b / 139b
13	148a / 153a / 147a / 150a / 142a
14	140b / 139b
15	146b / 148b / 143b
16	150a / 142a
17	156a
18	135b / 143b
19	142a / 143a
20	143b / 135b
21	152b / 150b

[a] Colour palette: azure, 4f (α-spin); gold, 4f (β-spin); black, 5d and 6s (see Fig. S40). [b] Greater than 10% contribution to transition. Orbital composition is given in Table S9.

Table S9 Sm Contribution to Acceptor MOs in **2-Br**

**MO**	**4f**	**5d**	**6p**	**6s**
140a	16.7	0.8	1.9	0.3
141a	97.9	0.2	0.2	0.0
142a	1.1	12.9	14.2	21.9
143a	1.0	27.0	9.2	6.1
144a	0.6	42.5	4.7	1.5
147a	0.4	29.6	21.9	4.0
148a	1.7	55.4	4.3	2.7
150a	1.4	30.3	6.2	1.2
153a	0.8	37.2	3.8	0.1
156a	0.3	26.0	5.7	0.1
138b	1.1	25.1	11.7	2.1
139b	1.4	29.6	18.6	2.7
140b	1.0	25.7	3.9	3.2
143b	4.3	12.8	8.8	0.8
146b	2.6	30.4	4.5	1.9
148b	9.0	16.4	13.8	3.4
149b	53.5	3.5	1.4	0.3
150b	42.6	10.8	3.5	0.6
151b	13.9	17.2	1.1	2.6
152b	34.4	9.8	2.4	0.8
153b	17.0	10.1	2.5	0.2
154b	39.7	8.7	1.6	0.5
155b	23.7	7.3	2.9	0.1
156b	26.9	9.9	1.4	0.4
158b	32.2	11.0	5.7	1.0
159b	19.8	6.2	4.1	0.4
160b	16.6	17.4	4.4	0.7
162b	37.0	12.0	3.4	0.2
165b	36.8	14.3	3.3	0.3
167b	31.0	4.4	2.3	0.7
168b	18.4	4.9	2.7	0.7

Table S10 Acceptor Orbital Composition of TD-DFT Calculated Transitions in **2-I**

**Transition**[a]	**Acceptor MO**[b]
1	164a / 167a
2	168a / 171a
3	170a / 169a
4	166b / 167b
5	171b / 170b / 169b
6	177b / 176b / 173b / 168b
7	185b / 188b
8	186b
9	149a
11	144b / 145b
12	144b / 145b
13	151a
14	148b / 147b / 145b
15	146b / 147b
18	161a / 159a
19	149b
20	158b / 149b
21	163a
22	161b / 152b / 163b
23	153a / 152a
25	154a
30	162a / 154a / 155a
31	156a
43	158a / 160a / 166a / 162a
44	162b / 163b
45	164b

[a] Colour palette: azure, 4f (α-spin); gold, 4f (β-spin); black, 5d and 6s (see Fig. S41). [b] Greater than 10% contribution to transition. Orbital composition is given in Table S11.

Table S11 Sm Contribution to Acceptor MOs in **2-I**

**MO**	**4f**	**5d**	**6p**	**6s**
149a	2.6	10.7	1.7	1.9
151a	1.3	10.2	21.7	12.2
152a	0.6	32.9	25.9	0.5
153a	0.3	14.9	52.0	0.4
154a	1.3	19.1	23.9	12.1
155a	0.2	29.0	18.4	0.5
156a	0.8	43.2	16.4	0.4
157a	0.6	41.8	21.3	0.1
158a	0.9	60.4	3.1	0.8
159a	0.5	50.1	4.1	1.0
160a	0.8	60.6	0.8	0.1
161a	1.1	54.0	2.3	0.8
162a	1.0	12.8	9.6	12.7
163a	4.4	33.4	5.1	0.3
164a	16.9	11.6	2.6	0.1
166a	8.5	14.6	3.0	0.4
167a	22.5	11.9	4.0	0.3
168a	55.6	1.9	1.1	0.1
169a	21.9	14.2	3.8	0.5
170a	41.7	6.5	1.2	1.1
171a	29.8	6.8	1.5	1.6
144b	3.4	12.4	1.6	2.4
145b	1.4	15.9	3.4	2.7
146b	1.4	10.5	19.1	11.9
147b	2.1	11.7	2.1	1.4
148b	2.1	6.7	1.6	2.3
149b	1.1	12.8	2.7	1.9
152b	1.4	14.9	26.8	13.5
158b	0.6	38.8	1.5	1.6
161b	8.3	26.4	2.5	0.1
162b	1.1	11.3	3.7	0.7
163b	5.1	12.1	3.4	0.6
164b	2.9	10.9	3.2	0.0
166b	22.8	11.7	0.6	0.6
167b	18.9	3.9	4.2	0.5
168b	25.4	5.7	1.0	0.6
169b	12.2	9.0	5.8	1.0
170b	6.9	9.1	5.1	0.1
171b	28.9	10.2	4.7	0.1
173b	21.0	9.0	2.7	0.6
176b	8.6	8.6	2.4	0.8

177b	27.6	7.3	3.2	0.4
180b	31.3	5.0	1.6	0.5
185b	27.1	7.2	1.9	0.3
186b	46.4	6.6	1.0	0.2

7. References

1. *CrysAlis Pro*, Agilent Technologies: Yarnton, England, 2010.
2. G. M. Sheldrick, *Acta Cryst. Sect. C.* 2015, **71**, 3.
3. O. V. Dolomanov, L. J. Bourhis, R. J. Gildea, J. A. K. Howard and H. Puschmann, *J. Appl. Cryst.*, 2009, **42**, 339.
4. L. J. Farrugia, *J. Appl. Cryst.*, 2012, **45**, 849.
5. *POV-Ray*, Persistence of Vision Raytracer Pty. Ltd.: Williamstown, Australia, 2004.
